# A Dimeric Protein-RNA Glue Promotes Multivalent eIF4A-RNA Assembly

**DOI:** 10.1021/acscentsci.6c00662

**Published:** 2026-08-01

**Authors:** Jie Liu, Megan K. Moore, Kevin Lou, Douglas R. Wassarman, Abolfazl Arab, Samuel Ojeda, Barbara Karakyriakou, Ann-Sophie Koglin, Christopher J. Ott, Luke A. Gilbert, Kevan M. Shokat

**Affiliations:** † Department of Cellular and Molecular Pharmacology, 8785 University of California, San Francisco, San Francisco, California 94158, United States; ‡ Howard Hughes Medical Institute, University of California, San Francisco, San Francisco, California 94158, United States; § Department of Chemistry, 43166 University of California, Berkeley, Berkeley, California 94720, United States; ∥ Quantitative Biosciences Institute, University of California, San Francisco, San Francisco, California 94158, United States; ⊥ Department of Bioengineering and Therapeutic Sciences, University of California, San Francisco, San Francisco, California 94158, United States; # Helen Diller Family Comprehensive Cancer Center, University of California, San Francisco, San Francisco, California 94158, United States; ∇ Department of Urology, University of California, San Francisco, San Francisco, California 94158, United States; ○ Arc Institute, Palo Alto, California 94304, United States; ◆ 2348 Massachusetts General Hospital Cancer Center, Charlestown, Massachusetts 02129, United States; ⬡ Department of Medicine, Harvard Medical School, Boston, Massachusetts 02115, United States

## Abstract

Ligand dimerization represents a powerful strategy to enhance avidity, potency, and selectivity. Leveraging the natural-product molecular glue rocaglamide (RocA), we identified BisRoc, a dimeric rocaglate ligand that potently and durably suppresses translation and exhibits greater specificity across a cancer cell line panel than the monomeric RocA. CRISPRi screening revealed that BisRoc activity is influenced by cellular context, including IFITM-mediated uptake, ABC-type efflux transporters, and the translation initiation factor eIF4A2. Mechanistic studies showed that the paralogs eIF4A1 and eIF4A2 are differentially sensitive to BisRoc-induced dimerization. Owing to the presence of multiple binding sites on RNAs, BisRoc-bridged eIF4A-RNA motifs assemble into higher-order complexes that promote stress-granule formation more efficiently than monomeric RocA. Given the widespread multivalency of RNA-RBP interactions, this ligand dimerization strategy may be extended to modulate the higher-order assembly of other RNA-binding proteins.

## Graphical abstract

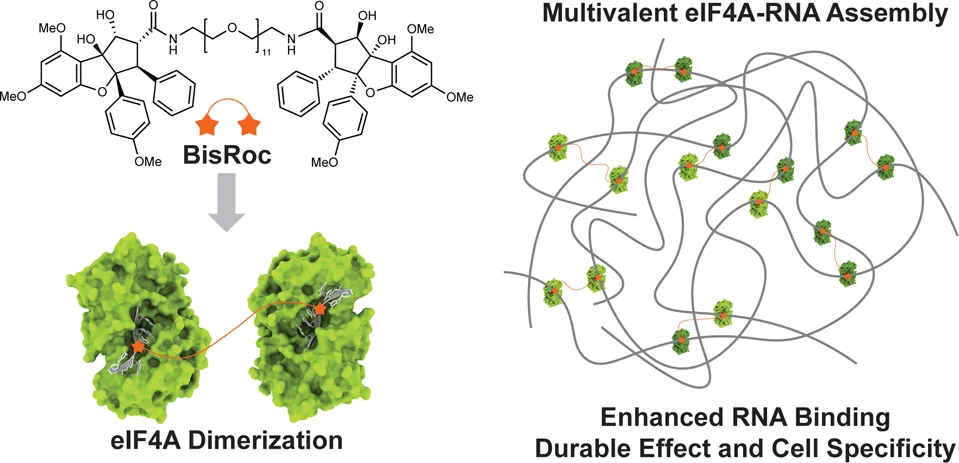

## Introduction

Eukaryotic translation initiation factor 4A1 (eIF4A1) is an ATP-dependent DEAD-box helicase which plays a critical role in 5′ mRNA cap-dependent protein synthesis.^1–3^ Multiple eIF4A1 molecules are thought to be recruited to 5′-untranslated regions (5′-UTRs) to unwind secondary structures and facilitate 43S preinitiation complex (PIC) scanning and translation initiation.^4–6^ Oncogenic transcripts such as MYC, Cyclin D1, and MCL-1 possess highly structured 5′-UTRs, rendering their translation sensitive to eIF4A1 availability.^7,8^ Elevated eIF4A1 expression has been observed in MYCN-amplified neuroblastoma, which underscores the reliance of certain tumors on this helicase.^9^ This dependency on eIF4A1 has motivated efforts to identify small molecules capable of modulating its activity.^10,11^

Rocaglates are a class of eIF4A1-targeting natural products including rocaglamide (RocA) that exhibits low-nanomolar potency across diverse cancer cell lines.^12–14^ Rocaglates act as molecular glues that clamp eIF4A1 onto polypurine (AG-rich) RNA motifs, thereby stalling protein synthesis.^15^ Ribosome profiling of RocA-treated cells together with eIF4A1 iCLIP data revealed multiple eIF4A1 binding sites across both 5′-UTRs and coding regions.^15,16^ These observations raise the question of whether increasing RocA valency could further enhance the potency or selectivity of eIF4A inhibition.

Ligand dimerization is a well-established strategy for increasing avidity and modulating protein activity.^17^ This approach has been successfully applied across diverse target classes, including cell-surface proteins,^18^ E3 ligases,^19^ inhibitor-of-apoptosis proteins (IAPs),^20^ transcription factors,^21^ and bromodomain and extra-terminal family proteins (BETs).^22,23^ For example, Bradner and co-workers showed that dimeric BET inhibitor MT1 is >100-fold more potent than the monovalent inhibitor JQ1.^22^ More recently, London, Levy, and colleagues demonstrated that dimeric ligands can further drive the polymerization of homomeric targets.^24^ Owing to the symmetric nature of these targets, dimeric ligands can amplify bivalency to multivalency. Considering the reported multivalent eIF4A1-RNA interactions,^6,16,25^ we hypothesized that rocaglate dimerization could promote higher-order eIF4A1-RNA assemblies, which might display activities distinct from monomeric rocaglates.^15^

In this work, we characterize the cellular activity of BisRoc, a dimeric rocaglate analog we recently reported in the context of the mechanism by which large molecular-weight molecules enter cells.^26^ By inducing eIF4A dimerization on RNA, BisRoc exhibits more sustained activity compared to the monomeric control following drug washout. A genome-wide CRISPRi screen identified the cellular import factor IFITM1 and the drug efflux pump ABCC1 as key gene dependencies consistent with the large molecular weight of the dimeric ligand. Unexpectedly, eIF4A2 exhibited a preferential contribution to BisRoc activity relative to RocA. Target engagement assays showed that BisRoc retained the canonical rocaglate target preference for eIF4A1 and eIF4A2, while promoting stronger cellular engagement of eIF4A2. In vitro experiments revealed distinct BisRoc-induced dimerization sensitivities of eIF4A2 versus eIF4A1 on RNA, driven by a single amino acid difference at the protein-RNA interface. Cellular assays showed that BisRoc promotes higher-order eIF4A-RNA assemblies. At higher concentrations, BisRoc further enhances condensate formation. Together, these findings reveal that a dimeric rocaglate bridges eIF4A-RNA motifs, driving higher-order assemblies that result in stronger and more sustained cellular activity.

## Results

### BisRoc’s Bivalency Confers Potent and Durable Translational Repression

Inspired by the multivalent interaction between multiple copies of eIF4A1 on a single mRNA, we designed a dimeric rocaglate, BisRoc, in which two rocaglate units were linked by a PEG11 spacer to enhance multivalent binding (Figure 1a).^26^ Previous studies have shown that rocaglates preferentially bind polypurine motifs.^15^ We therefore asked whether this enhanced multivalency could further confer sequence-selective inhibition of mRNA translation.^27^ To address this, we performed a puromycin incorporation assay to assess effects on global protein synthesis (Figure 1b).^28^ Puromycin serves as an indicator of protein synthesis that incorporates into the nascent peptides, leading to premature chain termination. Treatment with RocA robustly inhibited global protein translation in this assay (Figure 1b). The dimeric ligand BisRoc exhibited inhibitory activity comparable to RocA. Quantification of puromycin incorporation showed that the extent of translational suppression was consistent with the relative rocaglate activities observed in the cell viability assay (Figure 1c, Figure S1a).

**Figure 1:**
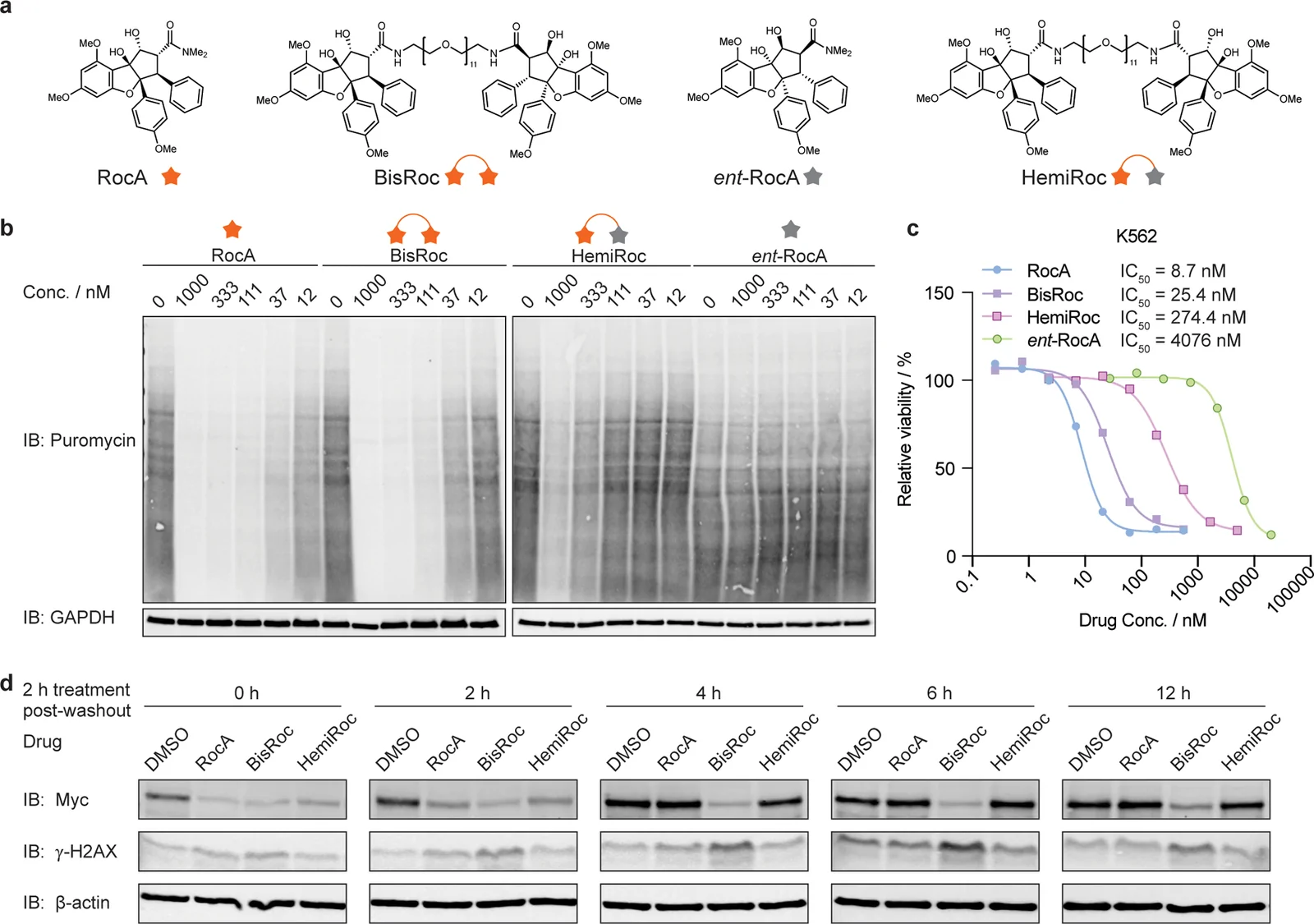
BisRoc exhibits potent and more durable translation suppression than HemiRoc. a. Chemical structures of RocA, BisRoc, *ent*-RocA, and HemiRoc. b. Puromycin incorporation assay comparing RocA, BisRoc, HemiRoc, and *ent*-RocA across a range of concentrations in K562 cells. c. CellTiter-Glo viability assay performed in K562 cells for 72 h. Data are presented as mean for three independent replicates. d. Washout assay (1 μM for drug pretreatment) on K562 cells assessing the durability of effect on Myc and γ-H2AX.

To quantitatively compare RocA and BisRoc, we performed translatome proteomics using O-propargyl-puromycin (OPP) (Figure S1b).^29^ After OPP incorporation, a biotin tag was introduced by copper-catalyzed click chemistry. The labeled nascent proteome was then enriched, digested, tagged by TMT10 reagents, and analyzed by quantitative mass spectrometry. RocA and BisRoc both exhibited highly correlated effects on nascent protein synthesis (Pearson *r* = 0.81), indicating largely overlapping sequence selectivity (Figure S1b).

To examine the similar sequence preferences of RocA and BisRoc, we performed an in vitro translation assay using synthetic luciferase mRNAs bearing distinct 5′-UTR repeat motifs, including AGA, CAA, and CCG (Figure S1c,d).^30^ Although BisRoc more strongly inhibited translation of luciferase bearing CAA- and CCG-repeat 5′-UTRs, the relative selectivity among the tested 5′-UTR sequences was largely unchanged: AGA remained the most sensitive motif, while CAA and CCG showed similar sensitivity. This result is consistent with our translatome proteomics analysis that RocA and BisRoc exhibited similar sequence selectivity (Figure S1b).

Given the similar nascent proteome selectivity of RocA and BisRoc, we next examined whether BisRoc’s bivalent architecture contributes to its cellular potency by generating HemiRoc, in which one rocaglate moiety of BisRoc was replaced by its inactive enantiomer, thereby disrupting bivalency but having the same molecular weight and atomic composition as BisRoc (Figure 1a). BisRoc has been shown to depend on interferon-induced transmembrane protein IFITM-mediated cellular uptake,^26^ likely due to its relatively large molecular weight. To test whether HemiRoc preserves this uptake-associated cellular sensitivity, we performed CellTiter-Glo assays in IFITM1–3 knockdown cell lines (Figure S2). Consistent with our design rationale, IFITM1–3 knockdown conferred resistance to both BisRoc and HemiRoc treatment. Therefore, the resulting HemiRoc serves as a functionally monomeric control, allowing us to study the specific contribution of the dimeric ligand.

*ent*-RocA has been reported as an inactive control for natural RocA,^31^ and this was confirmed in our puromycin incorporation assay, where no inhibition of protein translation was observed (Figure 1b). HemiRoc’s inhibition of global translation was reduced by more than 10-fold relative to BisRoc (Figure 1b, Figure S1a). We also evaluated the effects of these compounds on cell viability (Figure 1c). RocA and BisRoc strongly inhibited K562 viability, with half-maximum inhibitory concentrations (IC_50_) of 9 and 25 nM, respectively. Consistent with the puromycin incorporation results, HemiRoc exhibited markedly reduced activity, with an 11-fold loss of potency, whereas *ent*-RocA had no significant effect on cell viability at concentrations below 1 μM (Figure 1c). Together, these results support the conclusion that both active rocaglate moieties of BisRoc, which likely engage two target sites, mediate BisRoc’s potent cellular effects.

Because target dimerization can increase avidity and, consequently, target residence time, BisRoc’s activity was evaluated using a cellular washout assay. K562 cells were treated with 1 μM RocA, BisRoc, or HemiRoc for 2 h, after which the compounds were removed by replacing the cell medium. Cells were then cultured for the indicated times and pulsed with puromycin for 1 h to assess global translation (Figure S3a). At the starting point, protein translation was strongly suppressed by RocA, BisRoc, and HemiRoc. After 6 h of postwashout incubation, protein translation recovered to the DMSO level in RocA and HemiRoc pretreated cells, however, BisRoc pretreatment resulted in sustained suppression of protein synthesis. This sustained inhibition was still evident even after 12 h of postwashout incubation. Myc downregulation by BisRoc was also sustained for up to 12 h after washout, whereas Myc levels recovered to DMSO control levels within 4 h in RocA- and HemiRoc-pretreated cells (Figure 1d). A recent study by Irish and co-workers demonstrated that rocaglates trigger a robust DNA damage response in leukemia cells, characterized by elevated γH2AX levels.^32^ Consistent with this observation, BisRoc pretreatment in our system led to a prolonged increase in γH2AX that remained elevated for at least 12 h following compound washout (Figure 1d).

Myc mRNA levels were not correlated with Myc protein abundance (Figure 1d, Figure S3b), indicating that the observed Myc protein downregulation is not explained by reduced Myc transcript levels. Notably, relative Myc mRNA levels varied over the postwashout time course, whereas Myc protein abundance remained suppressed under BisRoc pretreatment condition. These findings support sustained BisRoc-mediated eIF4A-RNA complex stabilization and translational inhibition as the primary mechanism underlying Myc protein suppression. Moreover, Myc protein loss preceded robust γH2AX induction after washout (Figure 1d), suggesting that DNA damage signaling is more likely a downstream consequence of prolonged translational stress rather than the primary driver of Myc protein depletion.

Together, these results indicate that although BisRoc and RocA exhibit similar nascent proteome selectivity, BisRoc confers markedly prolonged functional suppression of translation following compound removal. This sustained activity is dependent on its bivalent architecture, as the monovalent control HemiRoc lacks comparable potency and durability.

### BisRoc Activity Is Influenced by eIF4A2 and Cellular Uptake/Efflux Pathways

To systematically characterize the cellular mechanisms mediating BisRoc-induced target dimerization, we performed an unbiased chemical-genetic screen to identify differential interactions with monomeric versus dimeric rocaglate molecular glues. CRISPR interference (CRISPRi) is a systematic genome-scale screening method.^33–35^ It not only reports on-target engagement but also reveals critical pathways underlying a drug’s mechanism of action. Using this approach, interferon-induced transmembrane proteins IFITM1–3 were previously identified as key proteins that promote the cellular uptake of large molecules, including BisRoc.^26^ Consequently, IFITMs were expected to emerge as hits in our CRISPRi screen for BisRoc.

The CRISPRi screen was conducted in pre-engineered K562 cells (i.e., K562 CRISPRi cells) that stably express CRISPRi machinery (Figure 2a). A genome-scale sgRNA library was transduced into K562 CRISPRi cells and selected with puromycin, after which the resulting cell pool was treated with DMSO, RocA (10 nM), or BisRoc (5 nM), respectively. Compound concentrations were selected based on stoichiometric considerations, with 5 nM BisRoc providing twice the molar concentration of rocaglate moieties compared to 10 nM RocA. During the first 3-day treatment, RocA and BisRoc exhibited comparable inhibition of cell growth (Figure S4a). Beyond Day 3, however, BisRoc consistently and more potently suppressed cell growth relative to RocA. To ensure sufficient cell numbers for sequencing analysis, BisRoc was removed on Day 7, though a sustained inhibition on cell growth was still observed. At the end of the screen, relative sgRNA abundance was quantified by next-generation sequencing (NGS) and gene-level phenotypes were determined using established methods.^34^

**Figure 2:**
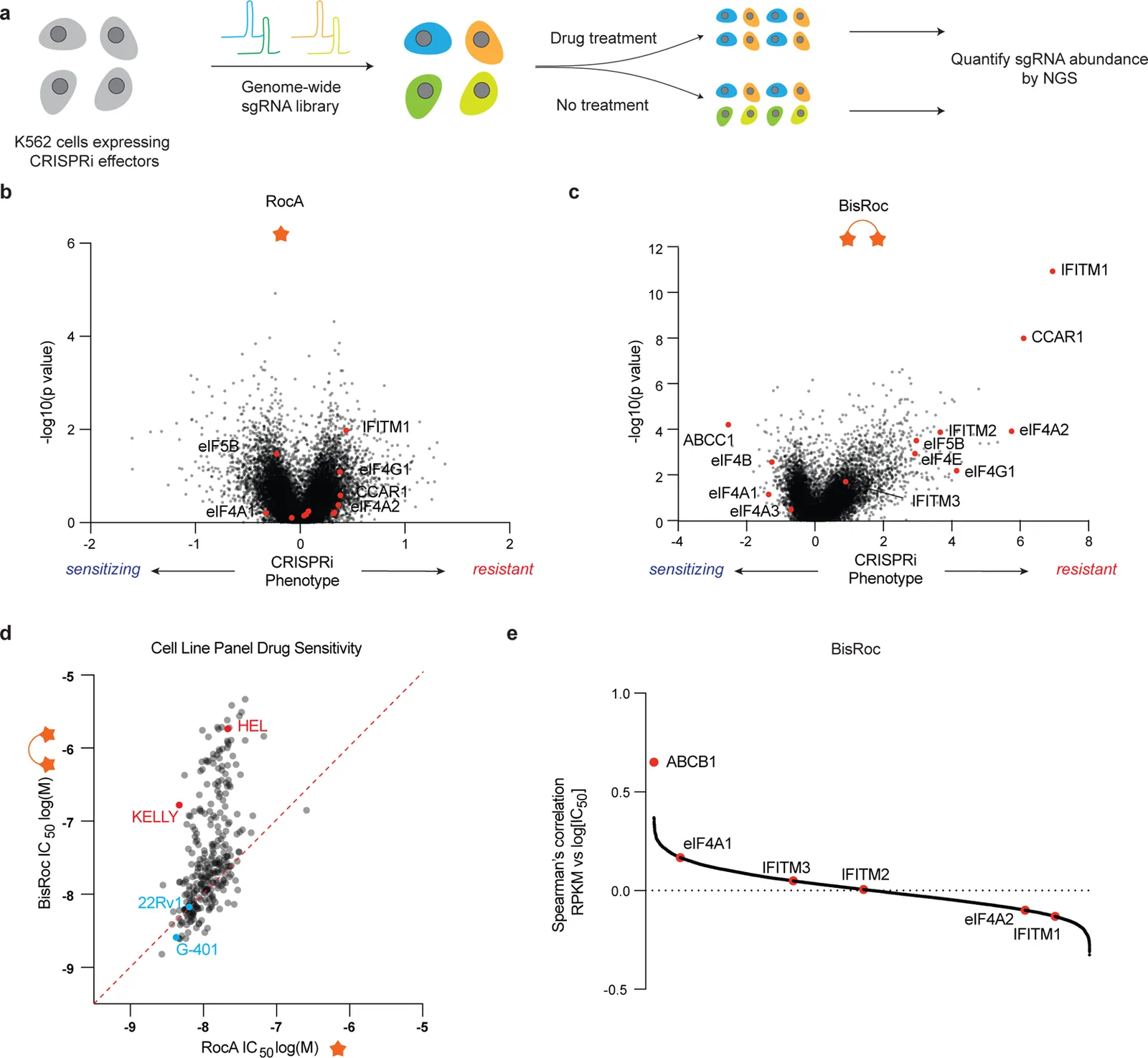
Functional genomics screening reveals distinct chemical-genetic interactions between RocA and BisRoc. a. Workflow of CRISPRi screening. b, c. CRISPRi screening results for RocA and BisRoc in K562 CRISPRi cells. d. Cell line panel screening results across 300 human cancer cell lines. e. Correlation of gene expression and BisRoc’s log­[IC_50_] values across the cell line panel.

RocA showed a relatively narrow distribution of gene-level phenotype scores, with 99.9% gene values ranging between −1 and 1 (Figure 2b). In contrast, BisRoc displayed a broader range of phenotypic scores across multiple genes (Figure 2c). This broader distribution may reflect both BisRoc’s distinct mechanism of action and the different selective pressures applied during the respective screens.^36^ As expected, BisRoc’s activity was highly influenced by the cellular uptake/efflux pathways, including IFITM1/2 and the ATP-binding cassette subfamily C member 1 (ABCC1) (Figure 2c). In our previous study, we discovered that IFITMs assist the cellular uptake of linked chemotypes, with IFITM knockdown conferring resistance.^26^ In contrast, ABCC1 functions as an efflux pump that exports molecules from the cytosol. Some proximity-inducing modalities such as PROTACs are reported to be sensitive to ABCC1 expression.^37^ Consistent with this, ABCC1 knockdown enhanced BisRoc sensitivity, suggesting that active cellular export mechanisms similarly regulate intracellular concentrations of linked chemotypes with long linkers (PEG11 in this case).

Cell Division Cycle and Apoptosis Regulator 1 (CCAR1) emerged as a resistance-associated hit in the BisRoc CRISPRi screen. CCAR1 was recently reported to regulate FANCA splicing and promote Fanconi anemia pathway function, such that CCAR1 loss sensitizes cells to DNA cross-linking agents.^38^ The opposite phenotype likely reflects the distinct selective pressure imposed by BisRoc versus DNA-damaging agents.

Notably, multiple translation initiation factors emerged as significant chemical-genetic interactors with BisRoc, including eIF5B, eIF4E, eIF4B, and eIF4G1 (Figure 2c). Rocaglates are reported to target eIF4A1 to impair protein translation.^14,15^ However, knockdown of eIF4A1 did not produce a significant sensitizing or resistant phenotype under either RocA or BisRoc treatment conditions, potentially as a consequence of its essentiality for cell growth. Unexpectedly, its paralog eIF4A2 emerged among the top resistance genes for BisRoc. We further validated a preferential contribution of eIF4A2 to BisRoc response by individually testing 3 sgRNAs targeting eIF4A2, all of which exhibited resistance to BisRoc treatment, showing an approximately 2-fold increase in IC_50_ upon partial knockdown (Figure S4b,c). The difference in the phenotypic magnitude between the focused Cell Titer Glo (CTG) assay and CRISPRi screen may reflect their distinct treatment formats. The CTG assay involved continuous treatment over 3 days, whereas the CRISPRi screen involved a longer selection period with repeated passaging and media replacement in the presence of compound. Because BisRoc induces durable translational suppression after washout, this longer screening format in CRISPRi screen may amplify modest differences in BisRoc sensitivity over time. Thus, cells with reduced eIF4A2 expression may become progressively enriched during the screen, whereas the shorter CTG assay captures only a modest resistance phenotype.

CRISPR screens are a powerful way to study a compound’s mechanism of action, but findings from individual cell lines may not be broadly representative. To complement our CRISPRi screen that was performed in a single cancer cell line, we also conducted a dose–response viability screen across a panel of 300 different cancer cell lines (Figure 2d).^39^ RocA demonstrated consistent cytotoxic potency across the panel, while BisRoc displayed substantial variability. For example, G401 and 22Rv1 cells showed comparable sensitivity to RocA and BisRoc. However, HEL and KELLY cells were more sensitive to RocA (Figure S5a-d).

To examine chemical-genetic interactions, we correlated IC_50_ values with transcript abundance across the cell lines (Figure 2e).^40^ High expression of ABCB1, an efflux pump, emerged as the strongest determinant of BisRoc resistance,^41^ which is analogous to our identification of ABCC1 as a hit in the CRISPRi screen and consistent with functional overlap among ABC transporters across different cellular contexts (Figure S5e). In addition, trends involving translation factors were observed but did not reach statistical significance (Figure 2e, Figure S5f,g). Notably, lower eIF4A2 expression was weakly associated with reduced sensitivity to BisRoc treatment, consistent in direction with CRISPRi results, though this correlation was modest (Figure 2e).

### BisRoc Binds with eIF4A1 and eIF4A2 in Cells

The identification of the preferential contribution of eIF4A2 to BisRoc activity in the CRISPRi screen attracted our attention as the eIF4A1 and eIF4A2 paralogs share 90% sequence identity. Both belong to the DEAD-box RNA helicases; however, eIF4A1 is expressed at much higher levels and serves as a core component of the eIF4F complex.^3^ Although recent reports have shown that eIF4A2 plays distinct roles in translational repression and stress response,^42–44^ it has generally been considered partially redundant to eIF4A1.^45,46^ Biochemically and functionally, rocaglates are reported to clamp eIF4A1 and eIF4A2 with comparable potency.^43,47^

The preferential contribution of eIF4A2 over eIF4A1 to BisRoc activity in our CRISPRi screen first suggested that BisRoc may exhibit a distinct paralog preference compared with RocA. To test this hypothesis, we needed to directly assess cellular target engagement for RocA and BisRoc. There have been several strategies to profile rocaglate targets, such as pull down,^48^ covalent labeling,^49^ and thermal shift proteomics.^50,51^ However, these methods are typically performed in lysates or require RNA/AMP-PNP (a nonhydrolyzable ATP analog) additives, making it challenging to assess target engagement in living cells.

To address this, we turned to photoaffinity-based proteomic profiling (Figure 3a).^52^ In the cocrystal structure of eIF4A1 with RocA and polyAG RNA, the amide side chain of RocA is solvent-exposed, providing a suitable site for chemical modification.^53^ We introduced a minimal photoaffinity group containing a terminal alkyne and diazirine at this site,^54^ yielding RocA-PAL (Figure 3b). This probe retained robust activity in K562 cells, as shown by CTG assays and Western blot analysis of Myc (Figure S6a,b). Upon UV irradiation, this probe forms covalent bonds with its cellular targets. To assess its photo-cross-linking capacity, we performed a dose-dependent labeling experiment in which the RocA-PAL labeled proteome was conjugated with TAMRA-azide (Figure 3c). At a concentration of 1 μM, RocA-PAL produced a relatively clean labeling pattern with a major band at around 55 kDa, which is consistent with the expected molecular weight of eIF4A1–3. Increasing the dose enhanced the intensity of this band, but other protein bands emerged as well. Based on these results, a concentration of 5 μM was selected for subsequent competitive photoaffinity-based proteomic profiling experiments to ensure sufficient labeling while minimizing off-target enrichment. RocA-PAL binding was competitively displaced during cotreatment with RocA or BisRoc at concentrations ranging from 5 to 50 μM, leading to reduced intensity of the band around 55 kDa, while other bands on the gel remained largely unaffected (Figure S6c).

**Figure 3:**
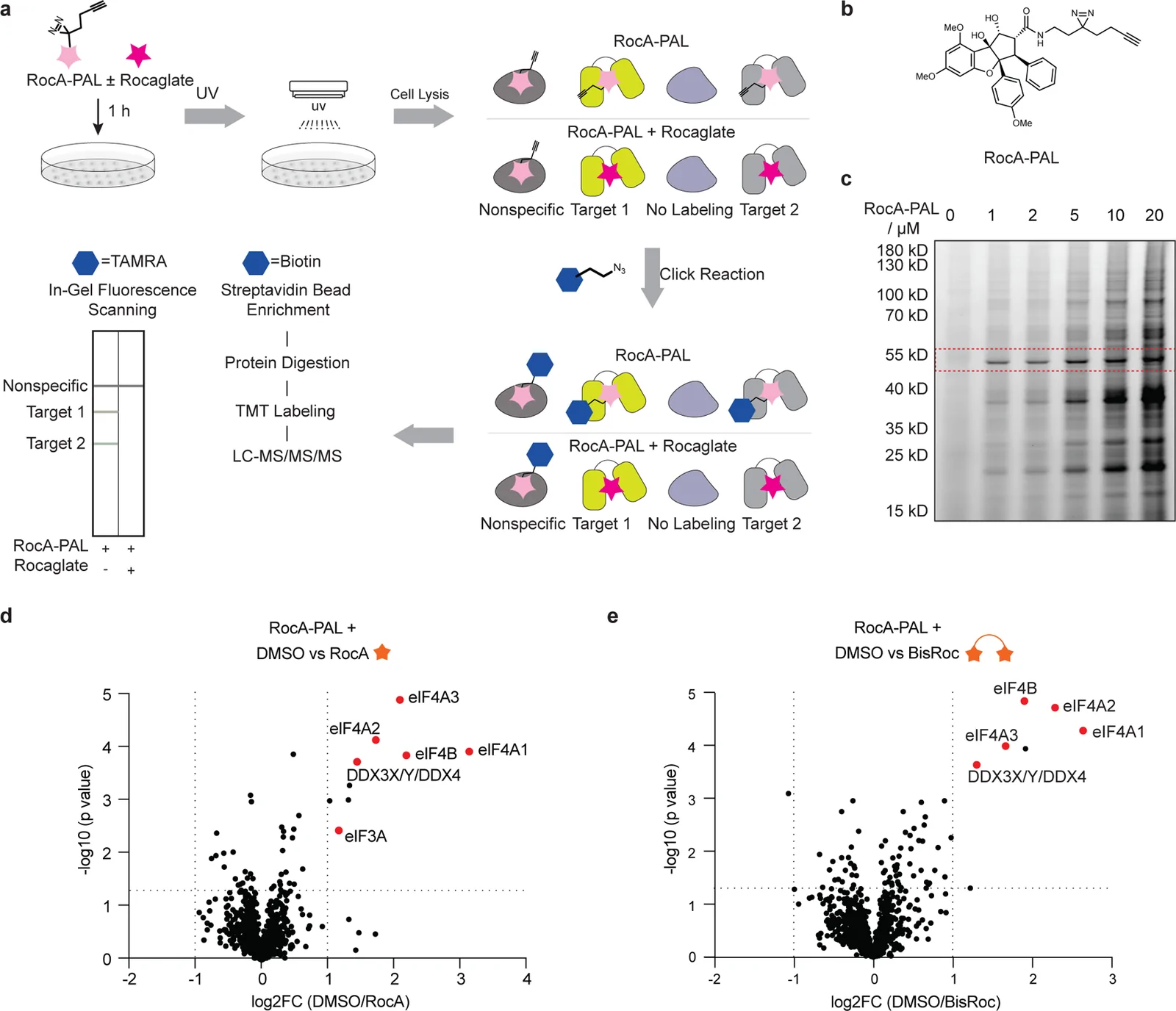
Target engagement evaluation of BisRoc using photoaffinity-based proteomic profiling assay. a. Workflow of photoaffinity-based proteomic profiling experiment. b. Chemical structure of RocA-PAL. c. TAMRA scan of photolabeled proteomes treated with increasing concentrations of RocA-PAL in K562 cells. d, e. Volcano plots from competitive photoaffinity-based proteomic profiling experiments using RocA or BisRoc as competitors in K562 cells.

To quantify target engagement, photolabeled proteomes cotreated with 50 μM competitors were conjugated with biotin-azide via click chemistry, followed by enrichment, digestion, TMT labeling, and quantitative proteomics. Consistent with previous reports on RocA targets,^48–51,55^ multiple helicases, including eIF4A1, eIF4A2, eIF4A3, and DDX3X/Y/DDX4, were identified in competitive photoaffinity-based proteomic profiling experiment using RocA or BisRoc as the competitor (Figure 3d,e). The translation initiation factor eIF4B also emerged as an interacting partner of rocaglates, likely due to its direct association with eIF4A1 during translation initiation, which may facilitate labeling by RocA-PAL. Although BisRoc did not alter the overall rocaglate target preference as both eIF4A1 and eIF4A2 remained engaged, it produced a modest shift toward eIF4A2 (Figure S6d).

One limitation of competitive photoaffinity-based proteomic profiling is that it requires relatively high concentrations of RocA and BisRoc. We therefore also employed the cellular thermal shift assay (CETSA), which measures the thermal stability of target proteins upon drug treatment, as an orthogonal method to assess target engagement at lower compound concentrations (Figure S7).^56^ K562 cells were treated with vehicle or 10 μM RocA or BisRoc and then heated across a temperature gradient. Drug binding is expected to alter the melting temperature of target proteins. Following lysis, soluble fractions were analyzed by Western blot against eIF4A1/2. Overall, CETSA results closely mirrored those from photoaffinity-based proteomic profiling, indicating that BisRoc retained engagement of both eIF4A paralogs, while producing a greater stabilization effect on eIF4A2 than RocA.

### eIF4A1 and eIF4A2 Exhibit Distinct Sensitivities to BisRoc-Induced Dimerization

When compared with its monomeric control HemiRoc, BisRoc exhibited substantially higher cellular potency (Figure 1c) and slower washout kinetics (Figure 1d), consistent with a dimerization-based mechanism. CRISPRi screening revealed a preferential contribution of eIF4A2 rather than eIF4A1. Although cellular target engagement assays indicated that BisRoc retained the canonical rocaglate target preference for both eIF4A1 and eIF4A2, they also suggested enhanced engagement of eIF4A2. Together, these findings motivated us to test whether eIF4A2 is more sensitive to BisRoc-induced dimerization. Although BisRoc’s binding specificity appears relatively unchanged, differential sensitivity to BisRoc-induced dimerization may explain the CRISPRi result and BisRoc’s enhanced activity relative to HemiRoc.

We used a band-shift assay in native gels to evaluate the dimerization efficiency of eIF4A1 and eIF4A2 using poly­[AG]_N_ RNA probes of varying lengths, 5′-labeled with 6-carboxyfluorescein (FAM). Under native gel electrophoresis conditions, the protein-RNA-drug complexes remain intact, and the FAM signal attached to the RNA serves as a readout of complex formation. In native gels, protein migration is primarily determined by size, shape, and net charge. When a protein binds negatively charged RNA, the resulting complex carries a higher net negative charge, which can cause it to migrate faster than the apoprotein despite its larger size. Furthermore, higher-order protein-RNA assemblies (containing either additional protein and/or additional RNA components) are up-shifted compared to monomeric complexes (i.e., a single protein bound to a single RNA). This approach allows us to directly compare the ability of BisRoc versus monomeric controls to promote multivalent eIF4A complexes on RNA substrates.

Incubating eIF4A2 and 5′FAM-[AG]_5_ with DMSO, RocA or HemiRoc produced a single band corresponding to a monomeric complex with 5′FAM-[AG]_5_ (Figure 4a, Figure S8a). Upon BisRoc treatment, a slower-migrating band appeared, which is consistent with our dimerization hypothesis. Intriguingly, BisRoc also induced the formation of a slower-migrating band for DDX3X (Figure S8b), another helicase reported to engage with rocaglates through a molecular glue mechanism.^49^ With the longer 5′FAM-[AG]_8_ or 5′FAM-[AG]_20_ RNAs and eIF4A2, BisRoc yielded a prominent slower-migrating band, whereas RocA and HemiRoc produced this band at lower abundance. Because longer RNAs provide multiple eIF4A binding sites, monomeric ligands can also support the formation of larger complexes, but these occur at substantially lower efficiency compared with BisRoc.

**Figure 4:**
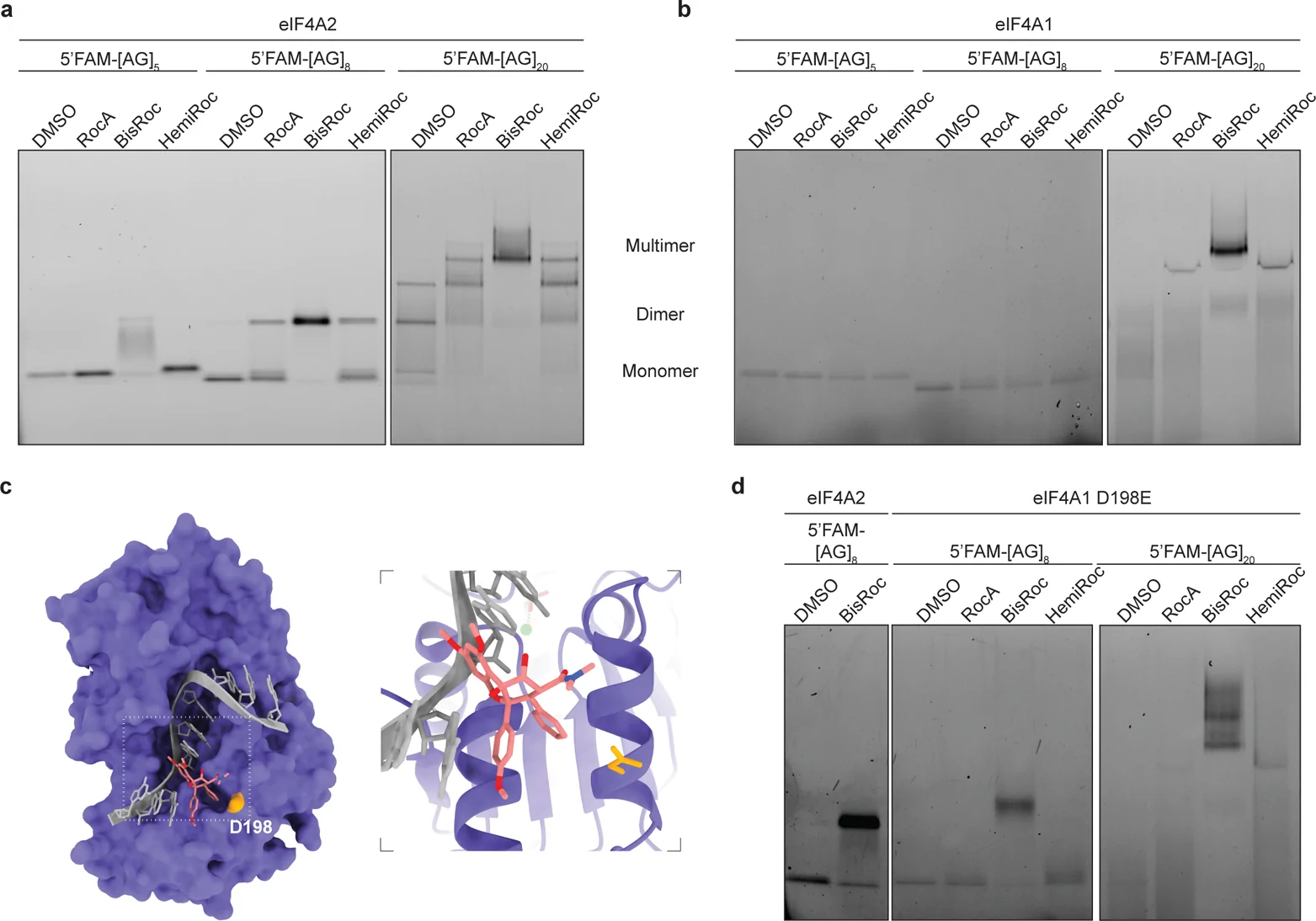
eIF4A2 is more sensitive than eIF4A1 to BisRoc-induced dimerization. a, b. FAM scan of native gel band-shift assay of eIF4A2 and eIF4A1 incubated with poly AG RNAs of varying lengths in the presence of different compound treatments for 30 min. 5′FAM-[AG]_5_ represents the 5′FAM labeled RNA sequence AGAGAGAGAG. Data are representative of two independent experiments. c. Cocrystal structures of eIF4A1-[AG]_5_-RocA (PDB: 5ZC9). Residue D198 is highlighted in orange. d. FAM scan of native gel band-shift assays for eIF4A1 D198E. The leftmost two lanes of the eIF4A2 condition were used as references for monomer and dimer bands. Data are representative of two independent experiments.

Since eIF4A1 and eIF4A2 possess nearly identical molecular weights, the bands corresponding to monomeric eIF4A2-[AG]_8_ (DMSO condition) and dimeric eIF4A2-[AG]_8_-BisRoc complexes (BisRoc condition) were used as references to calibrate complex sizes under other conditions. Incubation of eIF4A1 with [AG]_5_ or [AG]_8_ in the presence of compound yielded primarily a single monomeric complex (Figure 4b, Figure S8a). In contrast, when the longer [AG]_20_ RNA was used, BisRoc induced the formation of a much larger complex.

The native gel band-shift assay results are consistent with solution-based fluorescence polarization assay results using 5′FAM-poly­[AG]_N_ RNA as a probe (Figure S9a-f,i). When incubated with RNA probe and eIF4A2, BisRoc induced a striking increase in mP values across all different poly­[AG]_N_ regardless of length (Figure S9a-c). In contrast, when eIF4A1 was used instead, BisRoc and RocA produced nearly identical polarization signals with [AG]_5_ and [AG]_8_ (Figure S9d,e). When the RNA length was increased to [AG]_20_, we started to observe enhanced mP values upon BisRoc treatment, which suggests higher-order complex formation under such conditions (Figure S9f).

The observed different dimerization sensitivities of eIF4A1 and 4A2 despite their extremely high sequence similarity suggested that one or more nonconserved residues may drive this differential performance. Structural alignment of these two proteins revealed only one amino acid difference at the rocaglate-protein interface: D198 in eIF4A1 versus E199 in eIF4A2 (Figure 4c, Figure S9g). To test the role of this residue, we generated an eIF4A1 D198E mutant and tested it using the same fluorescence polarization assay (Figure S9h) and native gel band-shift assay (Figure 4d). Substitution of Asp198 with Glu resulted in increased mP values and the appearance of a distinct dimer band in the presence of eIF4A1 D198E-[AG]_8_-BisRoc, which phenocopied that by eIF4A2-[AG]_8_-BisRoc condition.

The D/E198 residue lies near the modeled composite interface formed by eIF4A, RNA, and BisRoc. Because BisRoc acts by stabilizing protein-RNA complexes, a residue at this position could influence the local electrostatic environment and the spatial orientation of the second rocaglate moiety within BisRoc, thereby affecting its ability to engage a second eIF4A-RNA complex. The RNA-length-dependent native gel band-shift results support this possibility: eIF4A2 forms BisRoc-induced higher-order complexes more readily, whereas eIF4A1 requires a longer RNA substrate, such as [AG]_20_, for detectable multimer formation.

### BisRoc Induces an RNA-Mediated eIF4A1-eIF4A2 Complex in Cells, Resulting in Protein-RNA Condensates

Since the rocaglate-eIF4A interaction is RNA-dependent, the tethering of two eIF4As by BisRoc would bring two RNA motifs together as well (Figure 5a). Given that multiple binding sites exist within a single transcript as well as across transcripts, BisRoc could create an eIF4A1/2-RNA network. To assess this mechanism, we employed a coimmunoprecipitation (CoIP) assay to first determine whether BisRoc induces the formation of such a complex.

**Figure 5:**
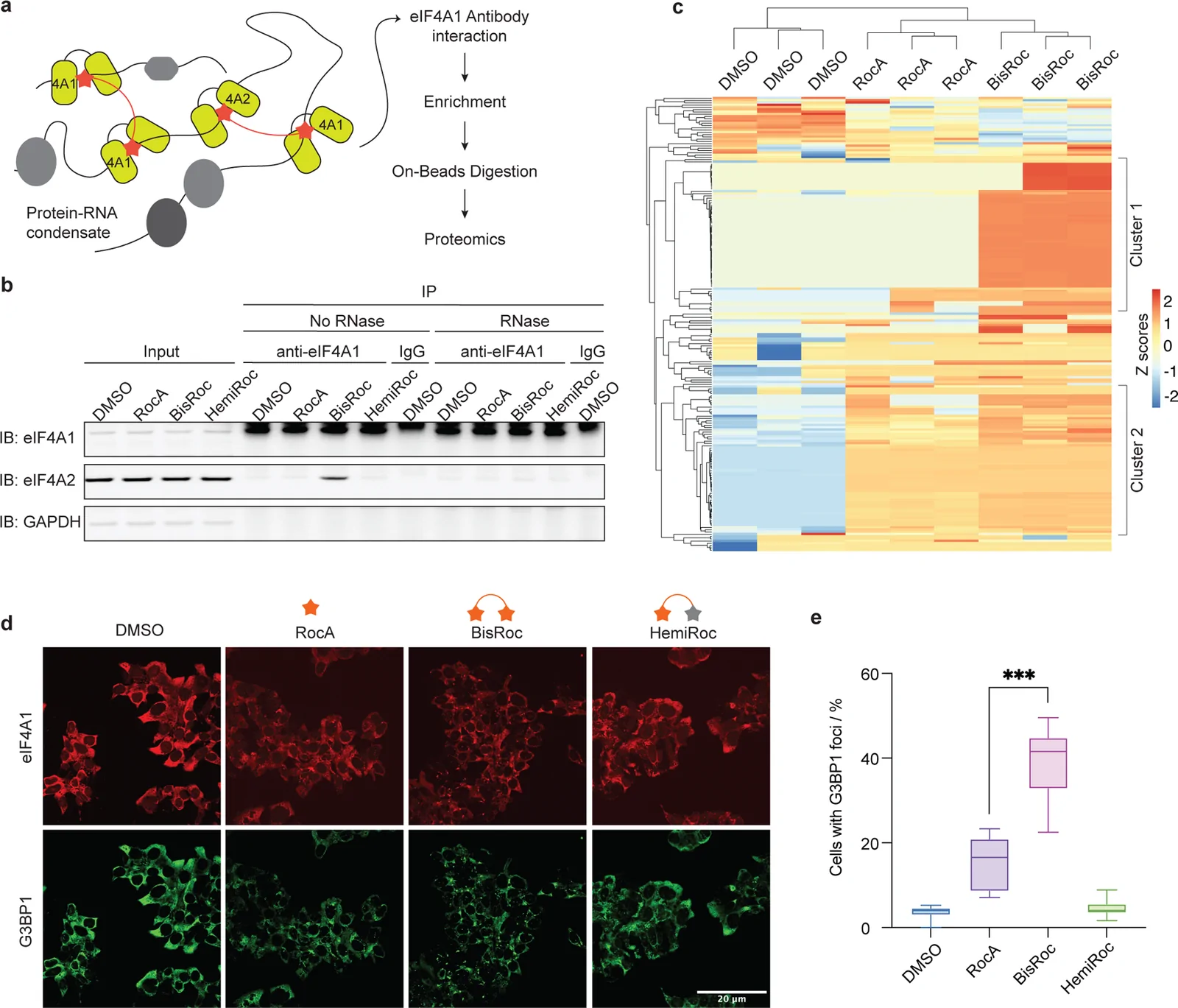
BisRoc promotes higher-order eIF4A-RNA assembly involving eIF4A1 and eIF4A2. a. Workflow of coimmunoprecipitation (CoIP) experiments using anti-eIF4A1 as the bait. b. CoIP results for different drug treatments at 2 μM concentration in K562 cells. Data are representative of two independent experiments. c. CoIP-MS heatmap in K562 cells showing three replicates for each condition. Two clusters were identified: proteins commonly enriched by RocA and BisRoc treatment; proteins uniquely enriched by BisRoc treatment. d. Immunofluorescence microscopy of cells treated with indicated drugs at 1 μM for 24 h in 22Rv1 cells. e. Quantification of 22Rv1 cells with G3BP1-positive foci. ***p < 0.001 by an unpaired two-tailed *t* test. Data are presented as mean ± s.d. (*n* = 3).

When the anti-eIF4A1 antibody was used as bait, eIF4A2 was coprecipitated under 2 μM BisRoc treatment, whereas no eIF4A2 enrichment was detected under vehicle-, RocA-, or HemiRoc-treated conditions (Figure 5b). This coimmunoprecipitation was RNA-dependent, as eIF4A2 was absent from the elutes upon RNase treatment. Similarly, the reciprocal experiment using an anti-eIF4A2 antibody also coprecipitated eIF4A1 under BisRoc treatment (Figure S10a). To investigate the dose-dependency of this phenotype, we performed CoIP experiments across a concentration range from 0.2 nM to 2 μM and found that BisRoc concentrations as low as 20 nM were sufficient to induce coprecipitation of eIF4A1 and eIF4A2 (Figure S10b). Together, these data support the conclusion that BisRoc induces RNA-mediated eIF4A1-eIF4A2 assembly in cells.

To define the composition of the BisRoc-induced eIF4A-RNA network, we performed a CoIP-MS experiment, in which the coprecipitated proteomes were digested and analyzed by mass spectrometry (Figure 5c). Unlike the CoIP/WB results, mass spectrometry did not capture the specific enrichment of eIF4A2 by BisRoc (Figure S10c). Upon closer inspection, we found that some peptides were misassigned by MaxQuant as eIF4A2-unique.^57^ Because eIF4A1 and eIF4A2 share extremely high sequence similarity, and because leucine and isoleucine are isomeric residues with identical mass, peptides differing only by I/L substitutions cannot be unambiguously distinguished by standard MS/MS analysis. In the region spanning residues 250–300, eIF4A1 and eIF4A2 differ only by a single I/L substitution (eIF4A1 I279 versus eIF4A2 L280; Figure S9g), generating tryptic peptides with identical molecular weight. After excluding the four ambiguous I/L-containing peptides, we observed that eIF4A2 enrichment was significantly higher under BisRoc treatment compared with vehicle or RocA (Figure S10d).

We next performed hierarchical clustering analysis of the CoIP proteomics data and identified two major protein clusters. Cluster 1 comprised proteins uniquely enriched by BisRoc and absent from both vehicle- and RocA-treated samples, whereas cluster 2 contained proteins enriched by both RocA and BisRoc relative to vehicle (Figure 5c). STRING cellular component GO analysis revealed that cluster 1 proteins are strongly enriched for cytoplasmic stress granule and ribonucleoprotein granule components, whereas cluster 2 is enriched primarily for more generic RNP complexes (Figure S10e,f).^58^ These data suggest that BisRoc promotes the accumulation of stress granule-associated ribonucleoproteins within the eIF4A-RNA network.

To validate this stress-granule association, we performed immunofluorescence microscopy to examine representative stress granule (SG) proteins, including G3BP1 and TIAR (Figure 5d, Figure S11a).^59^ BisRoc induced strong stress granule formation in approximately 40% of cells at 1 μM (Figure 5e), with G3BP1, TIAR, and eIF4A2 foci colocalizing with eIF4A1 (Figure S11a). In contrast, the monomeric control HemiRoc produced no significant signal, while RocA induced only weak SG formation at the same concentrations. When the concentration was increased from 1 μM to 20 μM, BisRoc displayed stronger SG-inducing activity (Figure S11b). Although RocA possesses comparable inhibitory effects on global protein translation as BisRoc (Figure 1b), its SG-inducing ability is significantly lower than that of BisRoc, indicating that enhanced SG induction by BisRoc cannot be explained simply by stronger translational repression, and instead is associated with its ability to promote higher-order eIF4A-RNA assemblies.^60^

## Discussion

Direct inhibition of oncogenic mRNA translation is a promising therapeutic strategy when selective suppression of a defined subset of transcripts is possible.^61^ Rocaglates enhance the affinity of eIF4A for polypurine sequences, thereby stalling protein translation by preventing 43S preinitiation complex (PIC) scanning.^15,49,62^ Although recent advances have improved selectivity for RNA motifs and target helicases, strategies to enhance transcript-level and cell-type selectivity remain limited.^27,51,63^

Here, we identified a dimeric rocaglate, BisRoc,^26^ which exhibits a greater cell-line-dependent activity than RocA across a cancer cell line panel (Figure 2d). Among the tested cell lines, K562 cells were highly sensitive to BisRoc treatment. We therefore selected K562 cells for genome-wide CRISPRi screening and most follow-up mechanistic studies, both because of the availability of the K562 CRISPRi platform and because this cell line represents a BisRoc-sensitive cellular context. This strategy allowed us to investigate the mechanism of BisRoc activity in a genetically tractable and pharmacologically responsive system.

Unbiased CRISPRi screening revealed that BisRoc’s activity is strongly dependent on cellular uptake and efflux pathways (Figure 2c). This dependency has also been observed in multiple proximity-inducing modalities.^26,37,41^ Large molecular-weight chemotypes often exhibit limited passive permeability, increasing their dependency on cellular uptake and efflux pathways. Notably, the CRISPRi hits were consistent with the dependency analysis derived from the cancer cell line profiling experiments, supporting a mechanistic basis for the cell-context-dependent sensitivity of BisRoc.

Furthermore, the CRISPRi screening unexpectedly revealed a preferential contribution of eIF4A2 to BisRoc activity relative to eIF4A1. eIF4A2 is generally considered functionally redundant with eIF4A1. Nevertheless, recent studies have shown that eIF4A2 is closely linked to translational suppression through its interaction with the CCR4-NOT complex.^42–44^ In this work, we show that the preferential contribution of eIF4A2 to BisRoc activity is associated with the greater propensity of eIF4A2, relative to eIF4A1, to undergo ligand-induced dimerization on RNA (Figure 3, Figure 4). Rocaglates act through a gain-of-function mechanism by stabilizing eIF4A-RNA complexes, leading to inhibition of translation. In this framework, the contribution of eIF4A2 to BisRoc activity may partly reflect a reduction in the total pool of rocaglate-responsive helicase-RNA complexes upon eIF4A2 depletion. Intriguingly, this sensitivity to dimerization is influenced by a single residue at the eIF4A-RNA-rocaglate interface. The D198E mutation in eIF4A1 increases sensitivity to dimerization relative to the wild type (Figure 4d).

Rocaglates are thought to inhibit translation through at least two mechanisms: clamping eIF4A onto polypurine-rich 5′-UTR elements to block 43S PIC scanning, and sequestering eIF4A in nonproductive complexes, thereby depleting it from eIF4F.^15,62^ Owing to its dimeric architecture, BisRoc may reinforce both processes by bridging two eIF4A-RNA motifs. Given the intrinsic multivalency of eIF4A-RNA interactions,^6,16,25^ BisRoc-mediated bridging may occur at multiple sites within a transcript or across distinct transcripts, thereby promoting higher-order polyvalent assemblies (Figure 5a). Consistent with this model, 20 nM BisRoc treatment was sufficient to induce coprecipitation of eIF4A1 and eIF4A2 in CoIP assays (Figure S10b), supporting a mechanism distinct from that of monomeric rocaglates. These multivalent eIF4A-RNA assemblies may also recruit additional RNA-binding proteins and help sustain stress granule formation. In agreement with this idea, immunofluorescence analysis showed that BisRoc induced stress granules more strongly than RocA (Figure 5d).

This work illustrates how dimeric ligand design can be leveraged to achieve context-dependent cell specificity. Multiple layers of cellular processes shape BisRoc activity: IFITMs influence uptake of large chemotypes, intracellular multivalent engagement stabilizes target assemblies and prolongs residence time, and ABC-type efflux pumps regulate compound export. Together, these factors create a selective landscape in which dimerization confers durable and context-dependent effects. More broadly, these findings demonstrate that dimeric molecular glue design can reorganize RNA-protein networks through multivalent engagement, revealing a mechanism distinct from monovalent clamping. Given the widespread multivalency of RNA-RBP interactions, rational ligand dimerization may represent a broadly applicable strategy for selectively modulating RNA-RBP networks with improved cellular selectivity.

## Supplementary Material





## Data Availability

All proteomic raw data have been deposited to MassIVE (http://massive.ucsd.edu) with accession no. MSV000101038, as well as in ProteomeXchange (http://www.proteomexchange.org) with accession no. PXD075236. Source data are provided with this paper. Code availability: Analysis code for OPP proteomics, photoaffinity-based proteomic profiling, and CoIP/MS is available on GitHub (https://github.com/LJ24601) under the project repositories OPP_proteomics, ABPP_rocapal, and coipms, respectively. CRISPRi screen analysis code is available via Zenodo (10.5281/zenodo.18870254).

## Notes

Source tables used to generate plots in the manuscript (ZIP)
Supplementary figures (Figure S1–S11), detailed experimental procedures for chemical synthesis and characterization, ^1^H NMR spectra for all compounds, and methods for all biological experiments, including OPP proteomics, in vitro translation, IFITM dependency, washout assays, CRISPRi validation, cellular response profiling, photoaffinity-based proteomics, CETSA, native gel band-shift assays, fluorescence polarization, coimmunoprecipitation, and immunofluorescence microscopy (PDF)
