# Supplementary material for: A Dimeric Protein-RNA Glue Promotes Multivalent eIF4A-RNA Assembly
Source: ACS Cent Sci. 2026 Aug 1;12(8):1195–206. doi: 10.1021/acscentsci.6c00662 (PMC13523671; doi:10.1021/acscentsci.6c00662)

Supporting Information for Publication

A Dimeric Protein-RNA Glue Promotes Multivalent eIF4A-RNA Assembly

Jie Liu[1,2], Megan K. Moore[1,2], Kevin Lou[1,2,4,5], Douglas R. Wassarman[1,2], Abolfazl Arab[1,2,5], Samuel Ojeda[9], Barbara Karakyriakou[9], Ann-Sophie Koglin[9], Christopher J. Ott[9-10], Luke A. Gilbert[6-8], Kevan M. Shokat[1-3*]

[1] Department of Cellular and Molecular Pharmacology, University of California, San Francisco, San Francisco, CA 94158, USA.
[2] Howard Hughes Medical Institute, University of California, San Francisco, San Francisco, CA 94158, USA.
[3] Department of Chemistry, University of California, Berkeley, Berkeley, CA 94720, USA.
[4] Quantitative Biosciences Institute, University of California, San Francisco, San Francisco CA, USA.
[5] Department of Bioengineering and Therapeutic Sciences, University of California, San Francisco, San Francisco, CA, USA.
[6] Helen Diller Family Comprehensive Cancer Center, University of California, San Francisco, San Francisco, CA 94158, USA.
[7] Department of Urology, University of California, San Francisco, San Francisco, CA 94158, USA.
[8] Arc Institute, Palo Alto, CA 94304, USA.
[9] Massachusetts General Hospital Cancer Center, Charlestown, MA 02129, USA.
[10] Department of Medicine, Harvard Medical School, Boston, MA 02115, USA.
* Corresponding author. Email: kevan.shokat@ucsf.edu (K.M.S.)

Supporting Information for Publication

Table of Contents

Supplementary Figures S3

Figure S1. OPP proteomics and in vitro translation assay. S3

Figure S2. IFITM dependency for BisRoc and HemiRoc. S4

Figure S3. Washout experiment measuring global translation efficiency by puromycin incorporation and mRNA levels by qPCR. S5

Figure S4. K562 CRISPRi cell growth during CRISPRi screen and validation of eIF4A2 dependency. S6

Figure S5. Validation of differential cellular responses to RocA and BisRoc. S7

Figure S6. Photoaffinity-based proteomic profiling experiment in K562 cells. S8

Figure S7. Cellular thermal shift assay (CETSA) for RocA and BisRoc. S9

Figure S8. Native gel band-shift assay for DDX3X. S10

Figure S9. Fluorescent polarization assay and sequence alignment of eIF4A1 and eIF4A2. S11

Figure S10. CoIP experiment for BisRoc treatment. S12

Figure S11. Immunofluorescence microscopy of cells treated with RocA or BisRoc at various concentrations in 22Rv1 cells. S13

Supplementary Tables S14

Table S1. CRISPRi sgRNA protospacer sequences S14

Table S2. Amino acid sequences of recombinant proteins S15

Table S3. List of antibodies S16

Chemical Synthesis and Characterizations S17

Synthesis of Roc-PEG11-amine S18

Synthesis of HemiRoc S18

Synthesis of RocA-PAL S19

Methods S20

Supplementary Reference S31

NMR Spectra S32

Purity Profile S35

Supplementary Figures

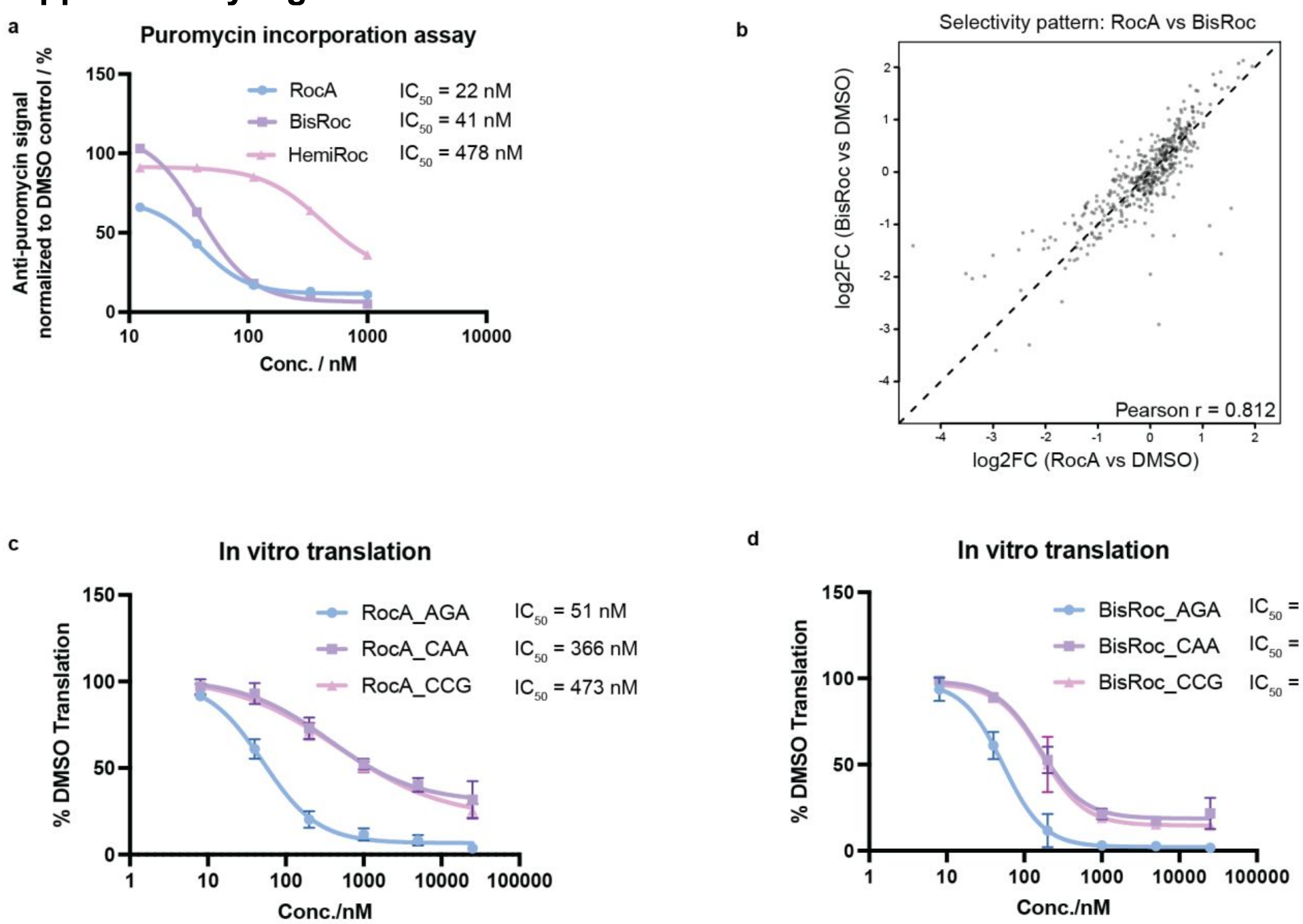

Figure S1. OPP proteomics and in vitro translation assay. a. Quantification of puromycin incorporation assay for Figure 1b. b. Selectivity analysis of nascent protein synthesis by OPP-based quantitative proteomics in K562 cells treated with 100 nM RocA or BisRoc, showing strong concordance in log2 fold-changes relative to DMSO (Pearson r = 0.812). c-d. In vitro translation assays using luciferase mRNAs bearing different 5'-UTR repeats. Data are presented as mean ± s.d. (n = 3). AGA represents 5'-UTR: 5′-AAGCTTGAA(AGAGAGCAACAA)6AGAGAGCACCAAGCTTGGCAATCCGGTACTGTTGGTAAAGCCACC-3′. CAA represents 5'-UTR: 5′-AAGCTTGAA(CAACAACAACAA)6CAACAACACCAAGCTTGGCAATCCGGTACTGTTGGTAAAGCCACC-3′. CCG presents 5'-UTR: 5′-AAGCTTGAA(CCGCCGCAACAA)6CCGCCGCACCAAGCTTGGCAATCCGGTACTGTTGGTAAAGCCACC-3′.

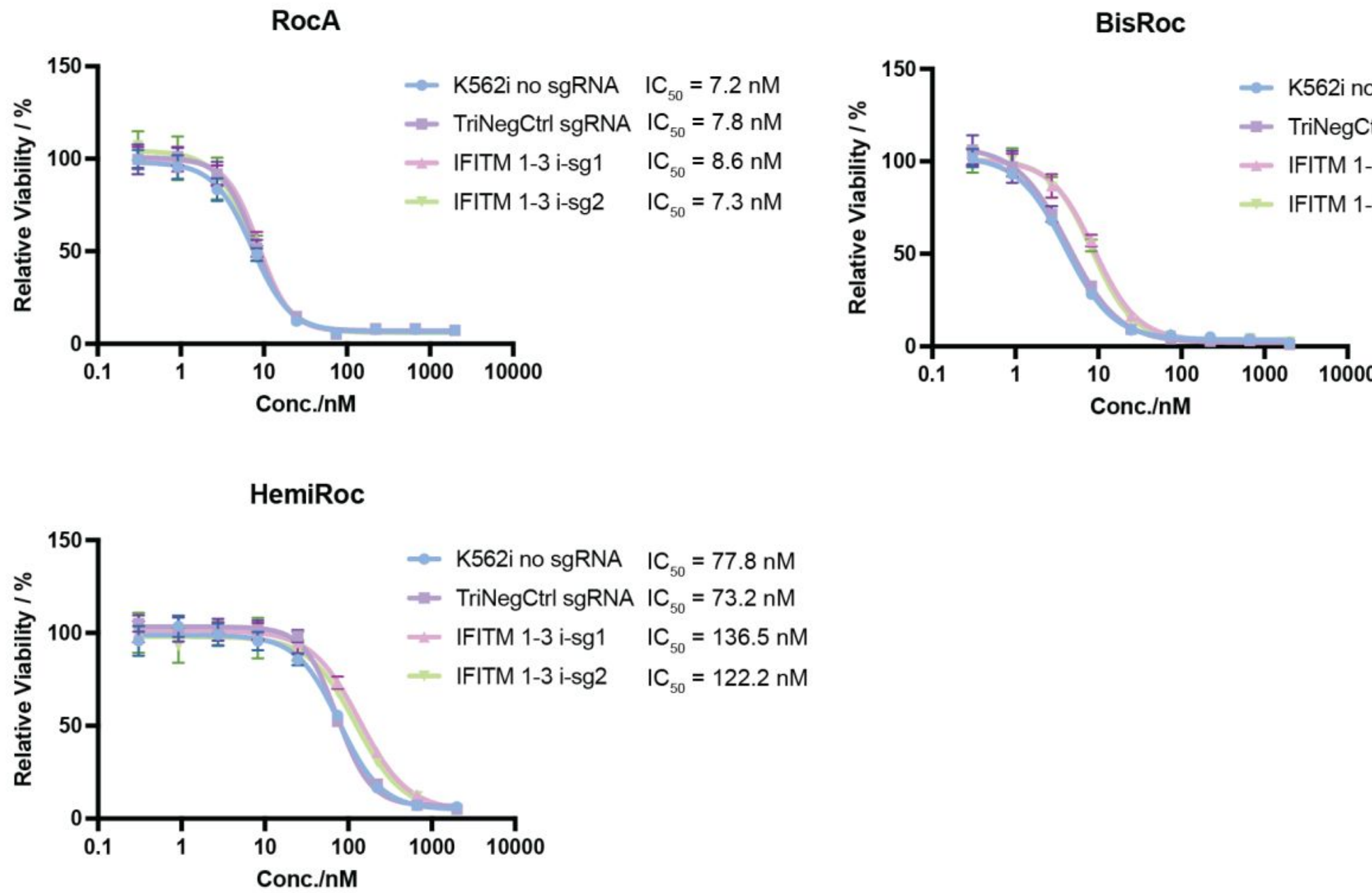

Figure S2. IFITM dependency for BisRoc and HemiRoc. The K562i IFITM1-3 sg1, IFITM1-3 sg2, TriNegCtrl cell lines are from previous work in our lab.[4] Data are presented as mean ± s.d. (n = 3).

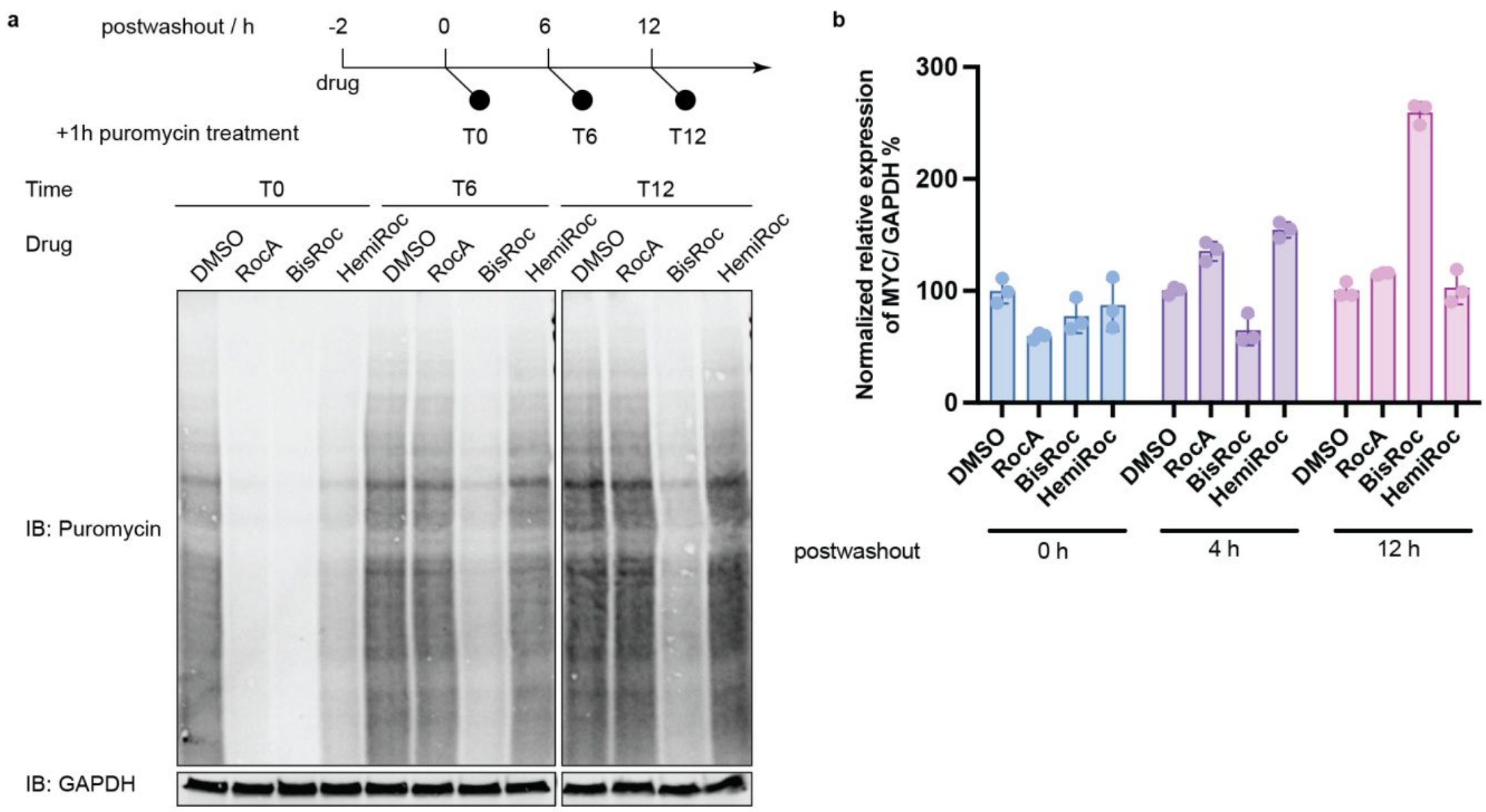

Figure S3. Washout experiment measuring global translation efficiency by puromycin incorporation and mRNA levels by qPCR. a. K562 cells were pretreated with 1 μM of indicated drugs for 2 h and washed three times with drug-free medium (5 min each), and then incubated for the indicated post-washout times. Puromycin was subsequently added and allowed to incorporate for 1 h before analysis. b. K562 cells were pretreated with 1 μM of indicated drugs for 2 h and washed three times with drug-free medium (5 min each), and then total RNAs were extracted for qPCR assays. Relative MYC mRNA levels were calculated using GAPDH as the internal reference gene and normalized to the DMSO control. Data are presented as mean ± s.d. (n = 3).

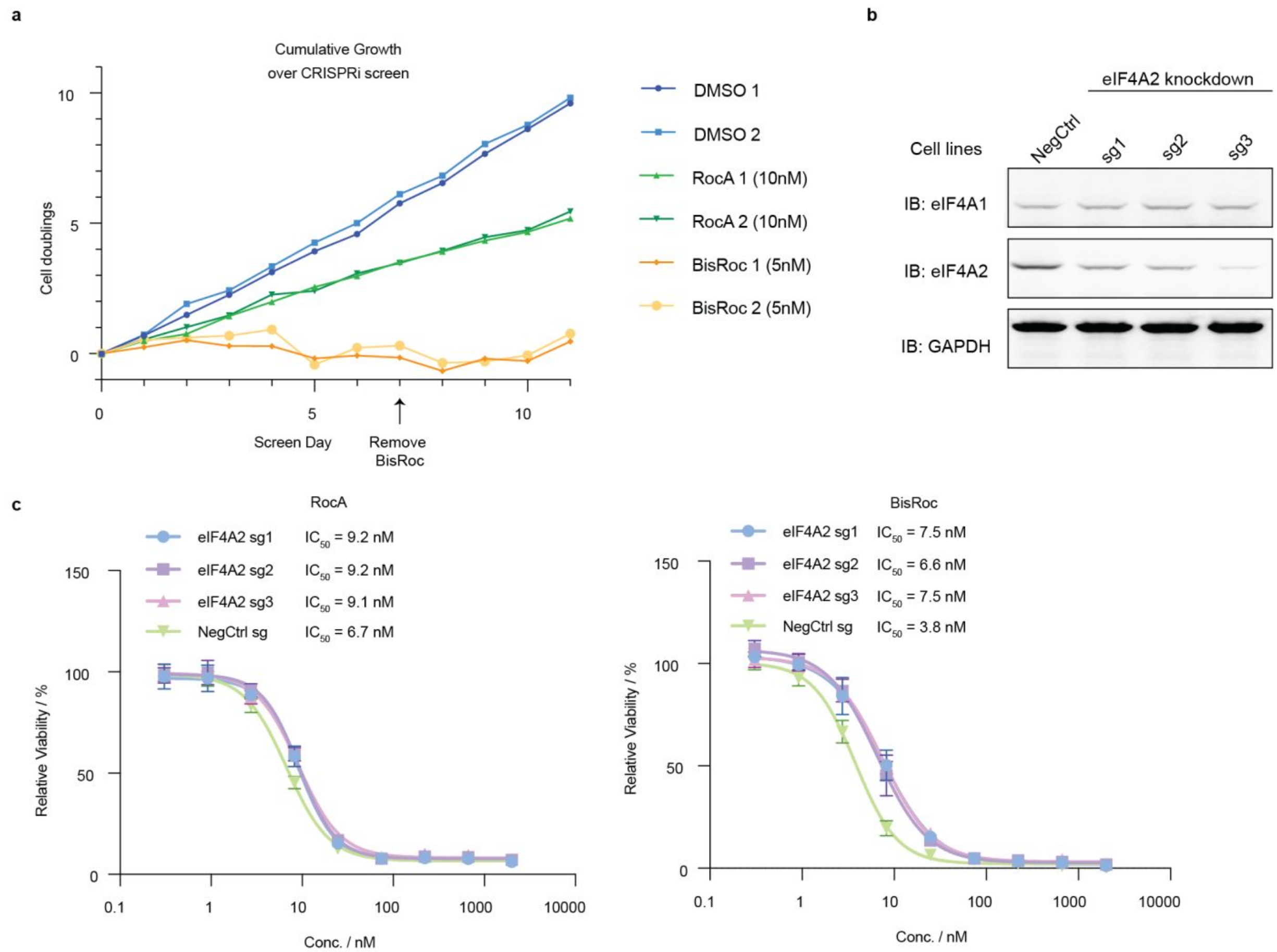

Figure S4. K562 CRISPRi cell growth during CRISPRi screen and validation of eIF4A2 dependency. a. Cumulative growth over the CRISPRi screen, shown for two independent replicates. b. Western blot detection of eIF4A1 and eIF4A2 levels in the generated stable eIF4A2 knockdown cell lines using sg1-3. c. Validation of eIF4A2 dependency by CellTiter-Glo viability assay performed on eIF4A2 knockdown cell lines (eIF4A2 sg1-3) or negative-control cell line. Data are presented as mean ± s.d. (n = 3).

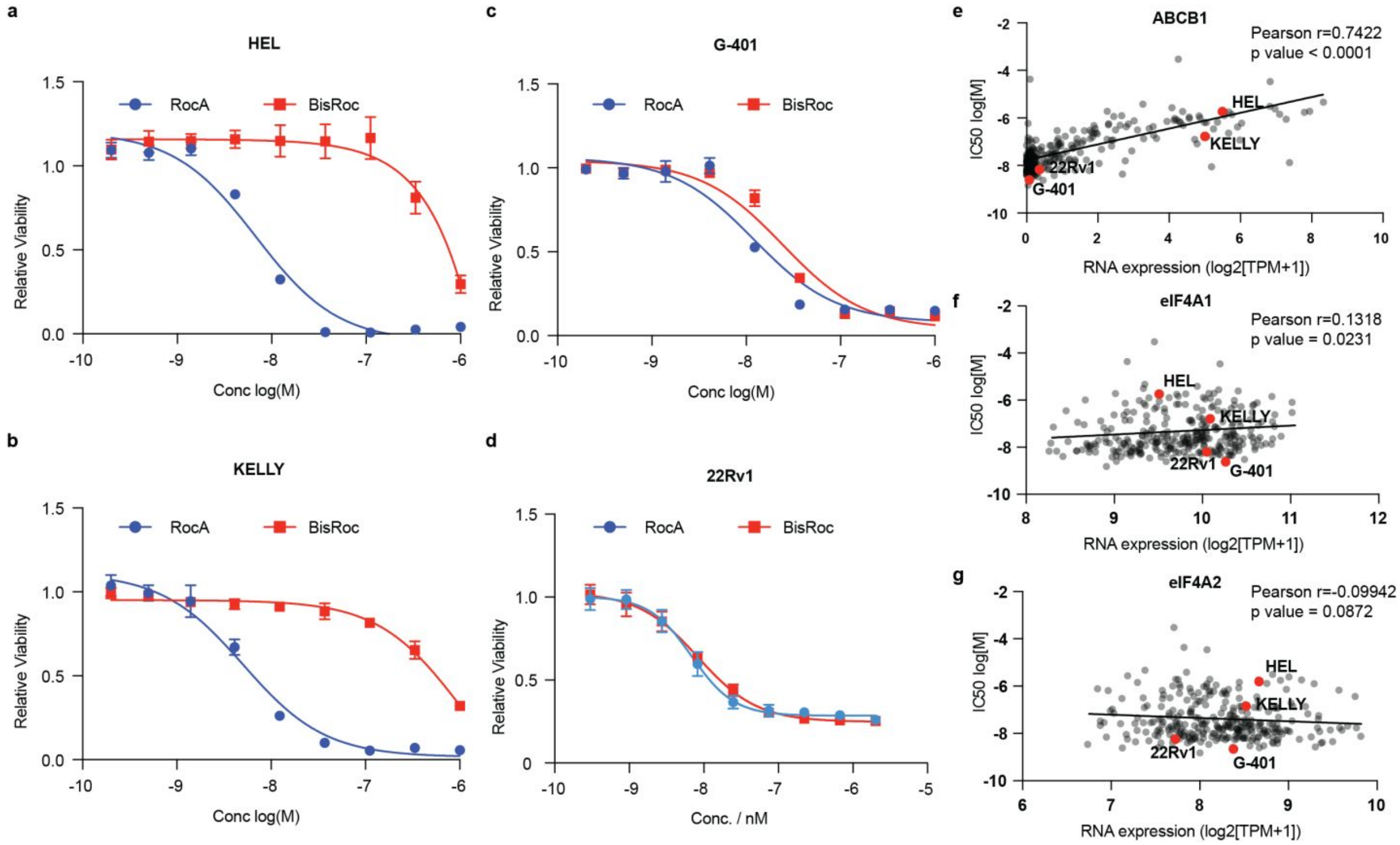

Figure S5. Validation of differential cellular responses to RocA and BisRoc. a-d, Cell viability measured by CellTiter-Glo (CTG) assays. Data are presented as mean ± s.d. (n = 3). e-g, Correlation between BisRoc IC_{50} values and transcript abundance of ABCB1, eIF4A1, and eIF4A2.

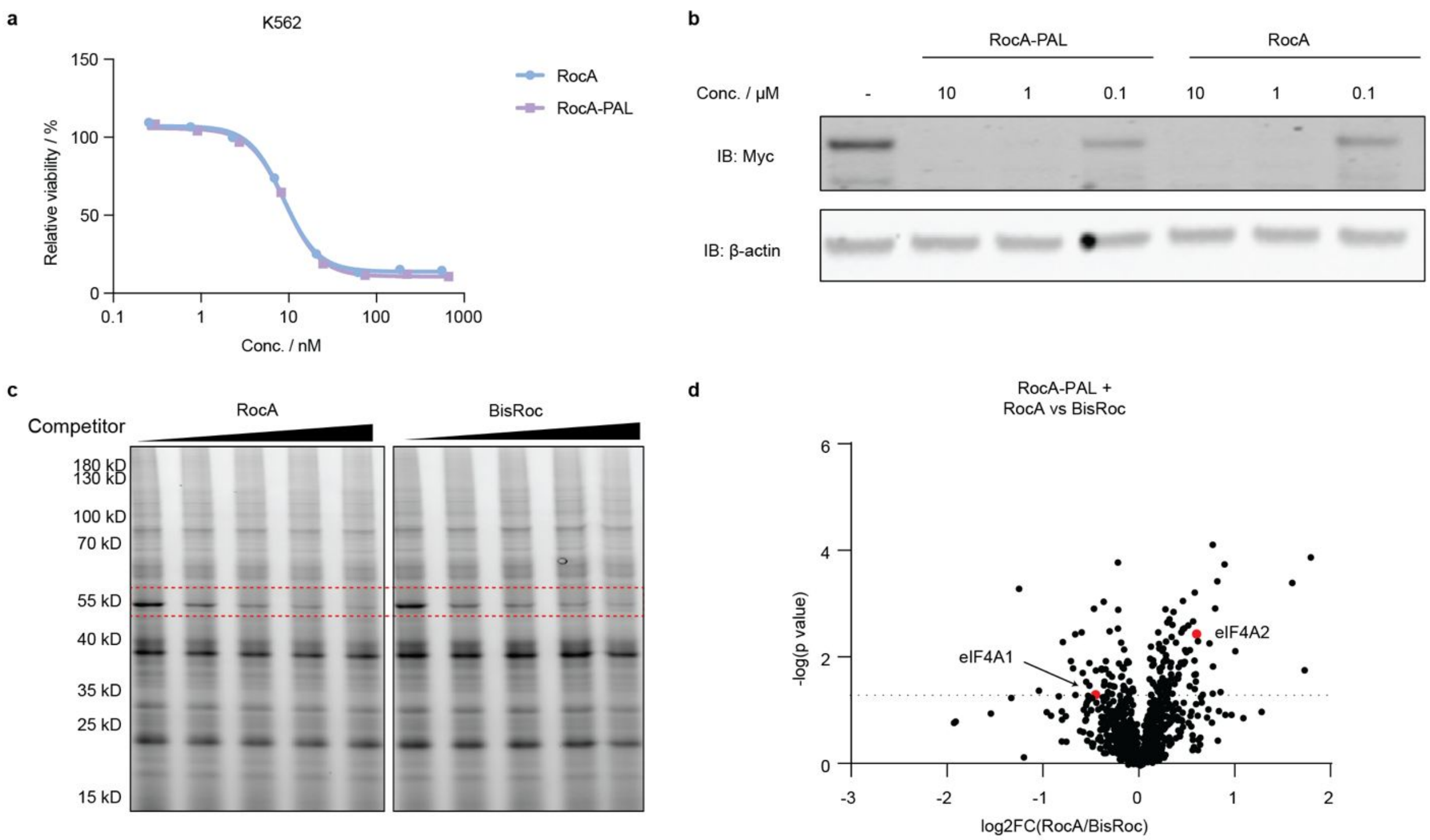

Figure S6. Photoaffinity-based proteomic profiling experiment in K562 cells. a, b. RocA-PAL possesses effects comparable to RocA on cell viability and Myc downregulation. Viability data are presented as mean of three independent replicates. c. TAMRA scan of proteome treated with RocA-PAL (5 µM) in combination with increasing concentrations of competitors RocA or BisRoc (0, 5, 10, 20, 50 µM) for 1 h. d. Volcano plot comparing protein enrichment between RocA and BisRoc competition conditions in the presence of RocA-PAL.

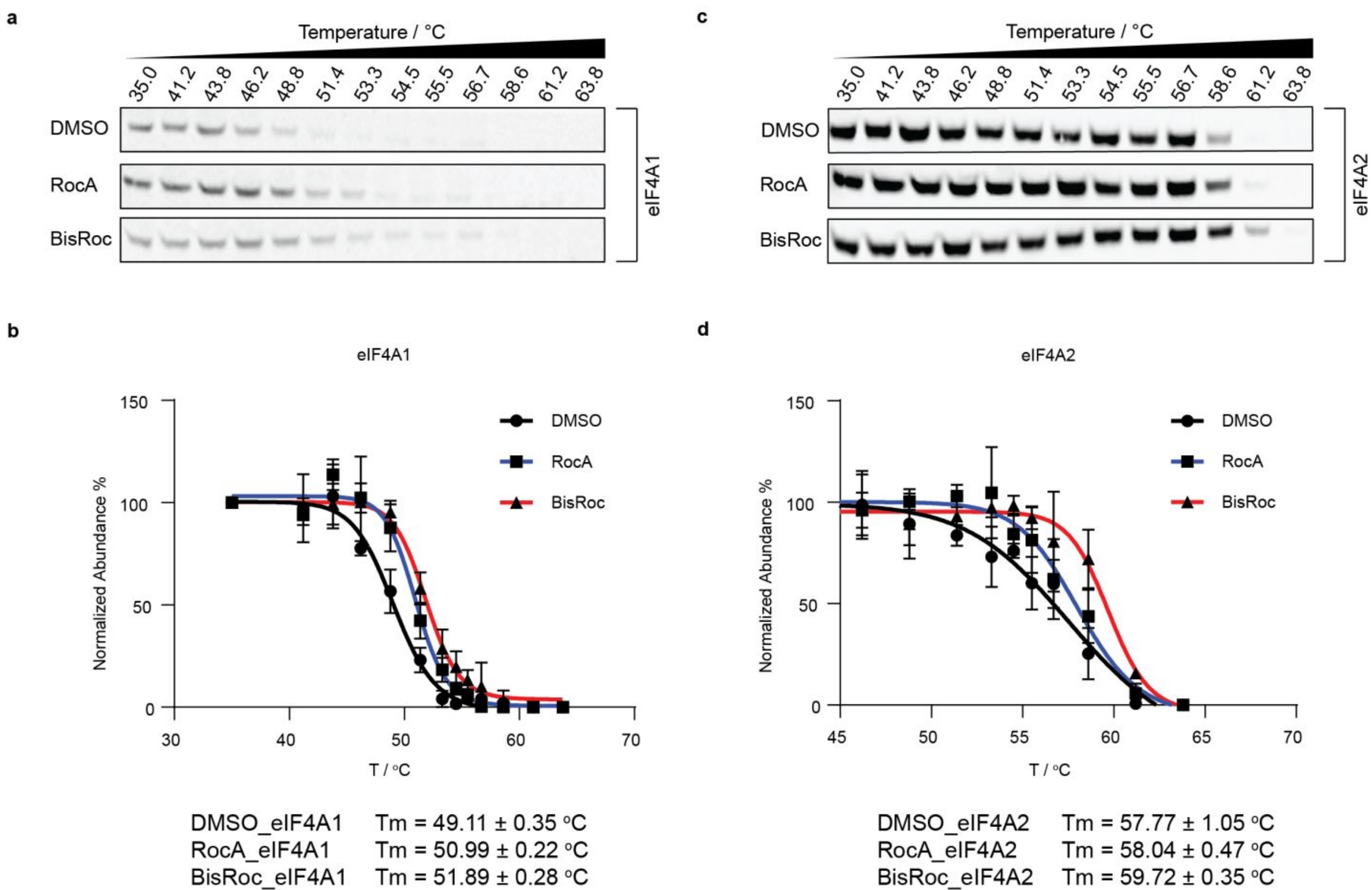

Figure S7. Cellular thermal shift assay (CETSA) for RocA and BisRoc. a, b. Western blot and quantification of CETSA results assessing the target engagement of eIF4A1. K562 Cells were incubated with indicated compounds (10 μM) for 1 h and then heated at the indicated temperature for 3 min. Data are presented as mean ± s.d. (n = 3). c, d. Western blot and quantification of CETSA results assessing the target engagement of eIF4A2 in K562 cells. Data are presented as mean ± s.d. (n = 3).

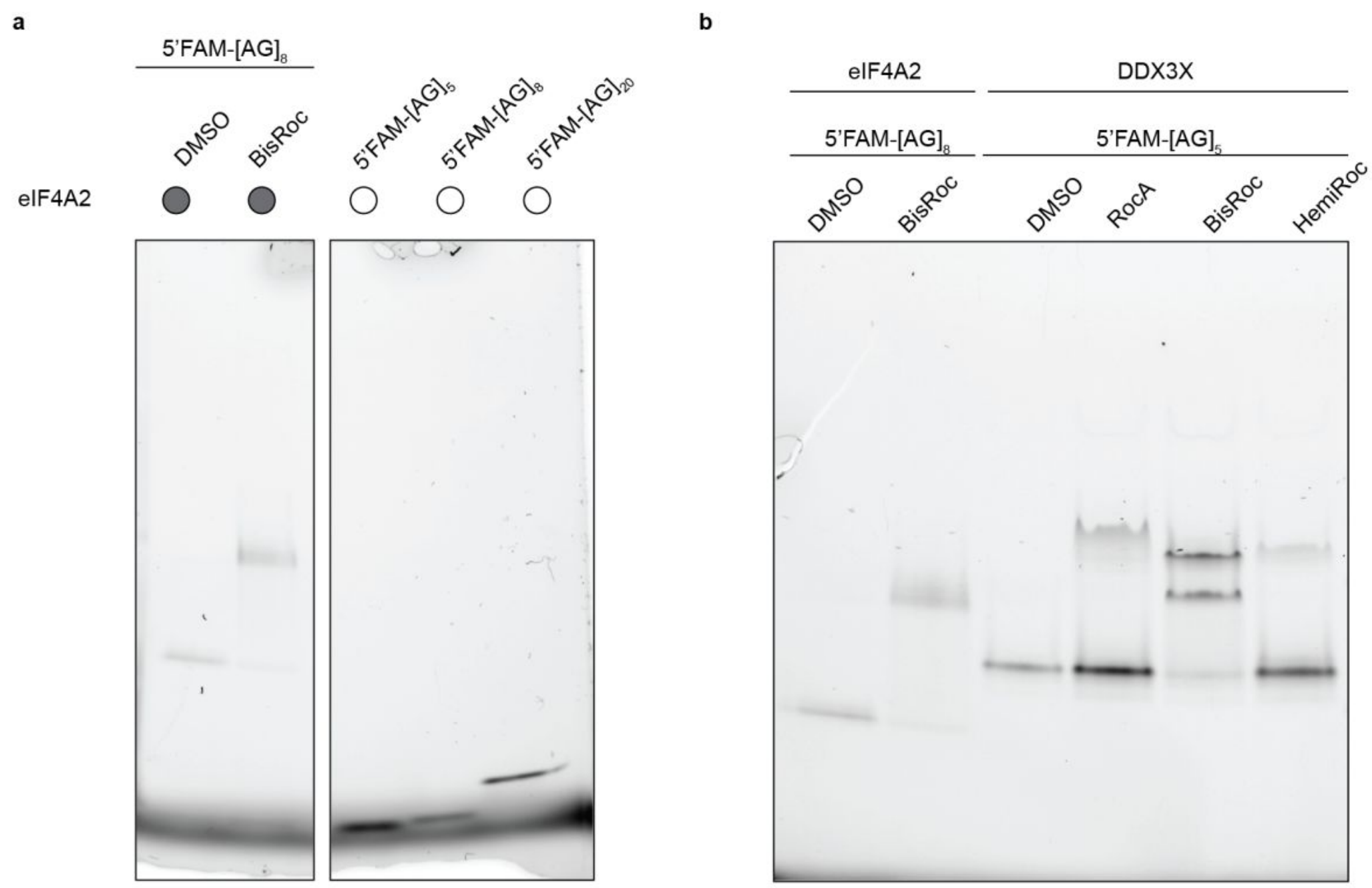

Figure S8. Native gel band-shift assay for DDX3X. a. The relative band location for 5'FAM-$[AG]_5$, $[AG]_8$, and $[AG]_{20}$ in the absence of proteins. b. FAM scan of native gel band-shift assay of DDX3X incubated with 5'FAM-$[AG]_5$ in the presence of different compound treatments for 30 min. The eIF4A2, 5'FAM-$[AG]_8$ in DMSO or BisRoc treatment was used as a reference control.

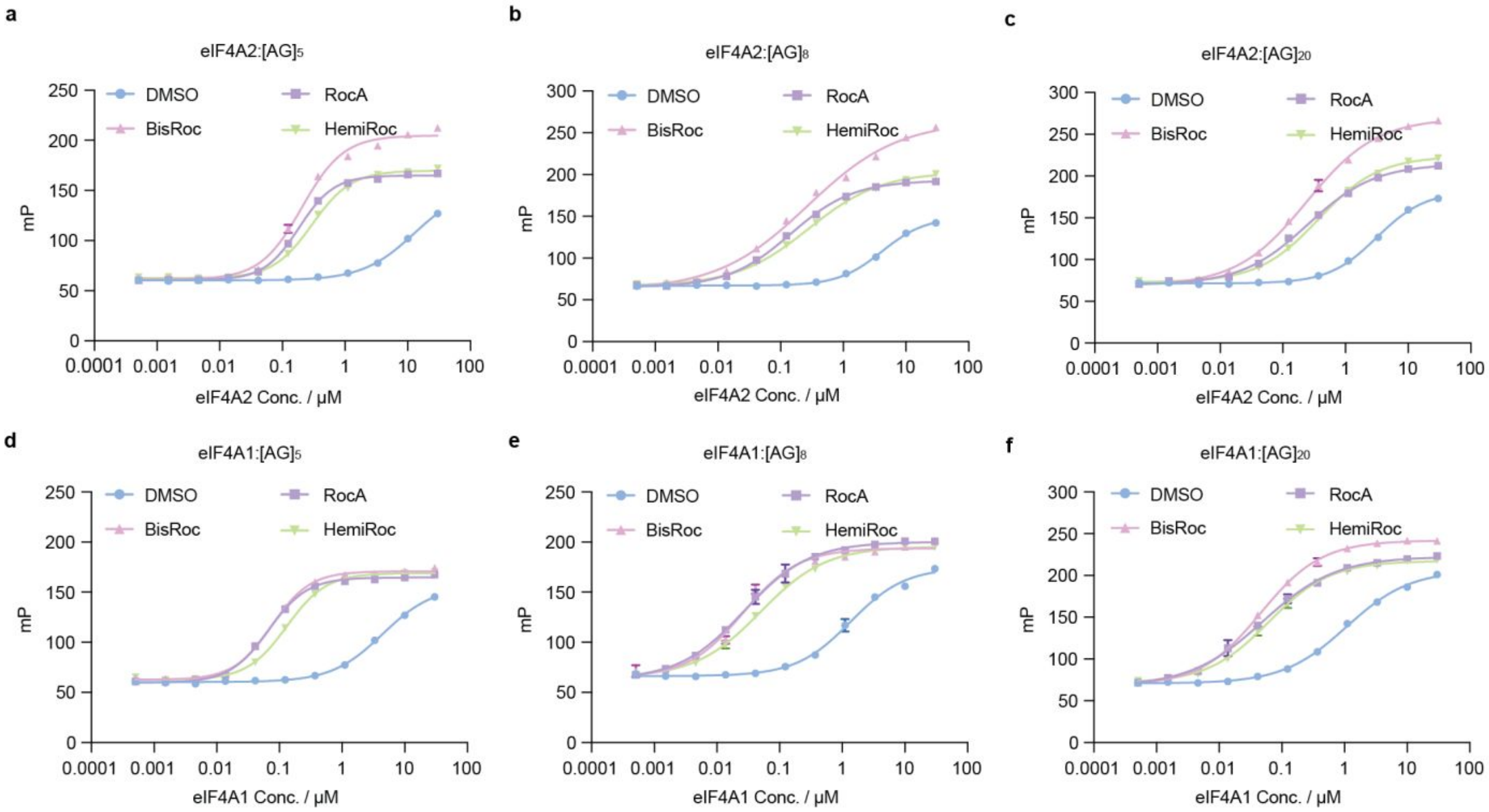

g

```
EIF4A2_HUMAN MSGGSADYNREHGGPEGMDPDGVIESNWNEIVDNFDDMNLKESLLRGIYAYGFEKPSAIQ 60
EIF4A1_HUMAN -MSASQDSRSRDNGPDGMEPEGVIESNWNEIVDSFDDMNLSESLLRGIYAYGFEKPSAIQ 59
               ..* * . ...**:**:*:*************.******.******************

EIF4A2_HUMAN QRAIIPCIKGYDVIAQAQSGTGKTATFAISILQQLEIEFKETQALVLAPTRELAQQIQKV 120
EIF4A1_HUMAN QRAILPCIKGYDVIAQAQSGTGKTATFAISILQQIELDLKATQALVLAPTRELAQQIQKV 119
             ****:*****************************:*:::* *******************

EIF4A2_HUMAN ILALGDYMGATCHACIGGTNVRNEMQKLQAEAPHIVVGTPGRVFDMLNRRYLSPKWIKMF 180
EIF4A1_HUMAN VMALGDYMGASCHACIGGTNVRAEVQKLQMEAPHIIVGTPGRVFDMLNRRYLSPKYIKMF 179
             ::********:*********** *:**** *****:*******************:****

EIF4A2_HUMAN VLDEADEMLSRGFKDQIYEIFQKLNTSIQVVLLSATMPTDVLEVTKKFMRDPIRILVKKE 240
EIF4A1_HUMAN VLDEADEMLSRGFKDQIYDIFQKLNSNTQVVLLSATMPSDVLEVTKKFMRDPIRILVKKE 239
             ******************:******:. **********:*********************

EIF4A2_HUMAN ELTLEGIKQFYINVEREEWKLDTLCDLYETLTITQAVIFLNTRRKVDWLTEKMHARDFTV 300
EIF4A1_HUMAN ELTLEGIRQFYINVEREEWKLDTLCDLYETLTITQAVIFINTRRKVDWLTEKMHARDFTV 299
             *******:*******************************:********************

EIF4A2_HUMAN SALHGDMDQKERDVIMREFRSGSSRVLITTDLLARGIDVQQVSLVINYDLPTNRENYIHR 360
EIF4A1_HUMAN SAMHGDMDQKERDVIMREFRSGSSRVLITTDLLARGIDVQQVSLVINYDLPTNRENYIHR 359
             **:*********************************************************

EIF4A2_HUMAN IGRGGRFGRKGVAINFVTEEDKRILRDIETFYNTTVEEMPMNVADLI 407
EIF4A1_HUMAN IGRGGRFGRKGVAINMVTEEDKRTLRDIETFYNTSIEEMPLNVADLI 406
             ***************:******* **********::****:******
```

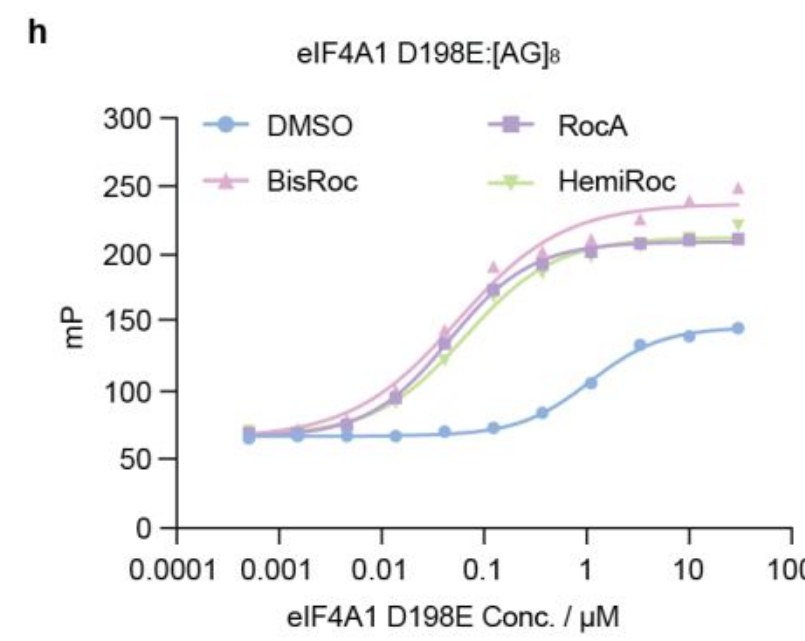

i

	eIF4A2 : [AG]5				eIF4A2 : [AG]8				eIF4A2 : [AG]20			
	DMSO	RocA	BisRoc	HemiRoc	DMSO	RocA	BisRoc	HemiRoc	DMSO	RocA	BisRoc	HemiRoc
EC50 / μM	12.93	0.18	0.20	0.30	4.15	0.14	0.27	0.28	3.16	0.25	0.24	0.37
Hillslope	1.04	1.56	1.30	1.38	1.25	0.87	0.60	0.75	1.13	0.81	0.78	0.90

	eIF4A1 : [AG]5				eIF4A1 : [AG]8				eIF4A1 : [AG]20			
	DMSO	RocA	BisRoc	HemiRoc	DMSO	RocA	BisRoc	HemiRoc	DMSO	RocA	BisRoc	HemiRoc
EC50 / μM	4.44	0.07	0.08	0.13	1.35	0.03	0.03	0.05	1.07	0.05	0.05	0.06
Hillslope	1.06	1.40	1.42	1.37	1.00	0.83	0.98	0.83	0.86	0.75	0.89	0.88

Figure S9. Fluorescent polarization assay and sequence alignment of eIF4A1 and eIF4A2. a-f, Fluorescent polarization assay using eIF4A2 or eIF4A1 at various concentrations and polypurine RNA sequences of varying lengths. Data are presented as mean ± s.d. (n = 3). g. sequence alignment of eIF4A1 and eIF4A2. h. Fluorescent polarization assay using eIF4A1 D198E at various concentrations and polypurine RNA sequence $[AG]_8$. Data are presented as mean ± s.d. (n = 3). i. Quantification of apparent EC50 and Hill slope values for panels a–f.

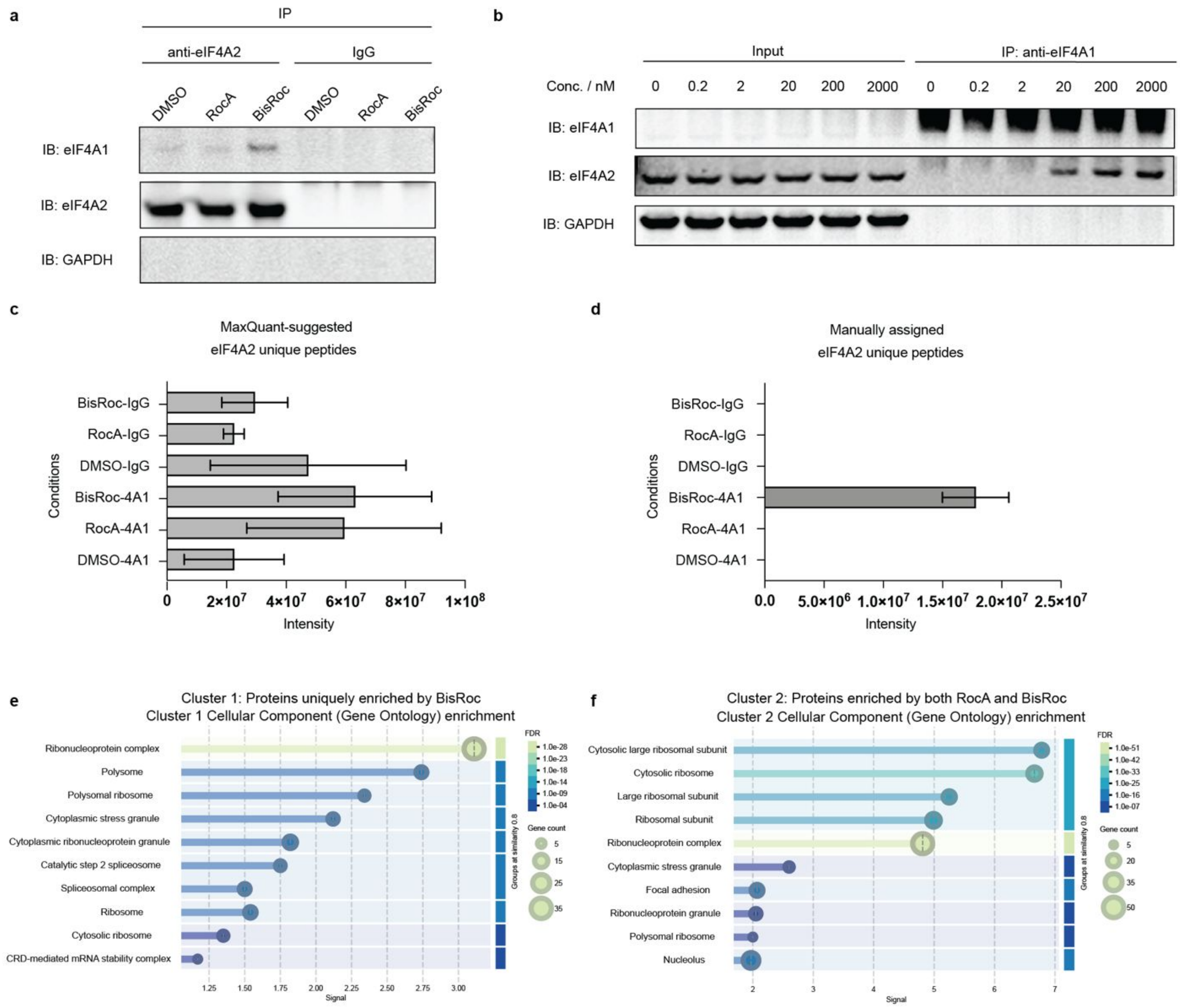

Figure S10. CoIP experiment for BisRoc treatment. a. anti-eIF4A2 co-immunoprecipitates eIF4A1 upon BisRoc treatment in K562 cells. b. Dose-dependent CoIP experiment across a gradient of BisRoc concentrations (2000 nM to 0 nM) in K562 cells. An anti-eIF4A1 antibody was used for immunoprecipitation. Data are representative of two independent replicates. c. Intensities of MaxQuant-identified eIF4A2 unique peptides. d. Intensities of manually assigned eIF4A2-unique peptides after excluding four I/L-containing peptides. e. Pathway analysis of the protein cluster 1 uniquely enriched by BisRoc. f. Pathway analysis of the protein cluster 2 commonly enriched by RocA and BisRoc.

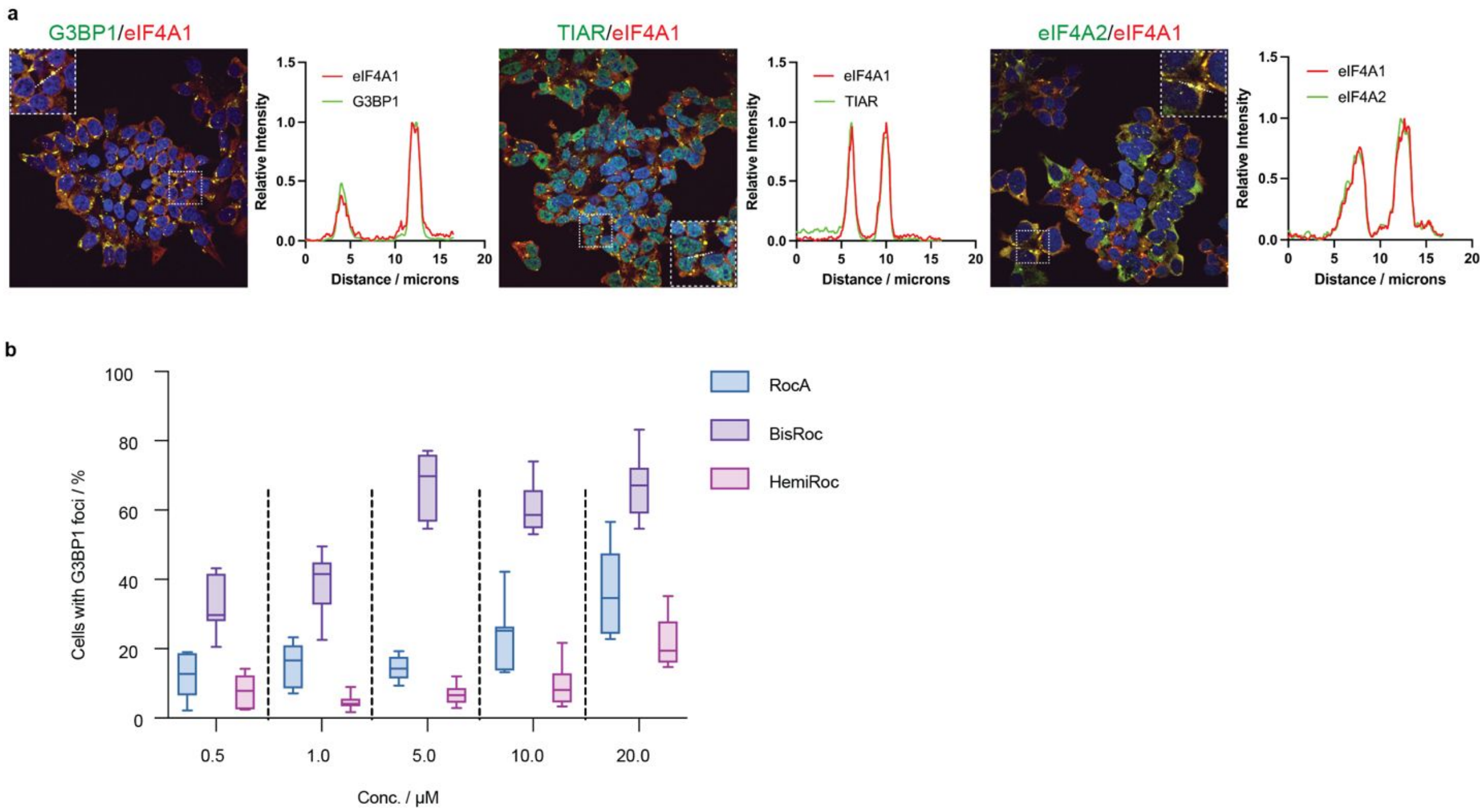

Figure S11. Immunofluorescence microscopy of cells treated with RocA or BisRoc at various concentrations in 22Rv1 cells. a. eIF4A1 co-localizes with G3BP1, TIAR, and eIF4A2 under the treatment of 1 μM BisRoc. b. Quantification of G3BP1 foci-positive cells across the range of drug concentrations tested. Data are presented as mean ± s.d. (n = 3).

Supplementary Tables
Table S1. CRISPRi sgRNA protospacer sequences

	Sequence
eIF4A2_sgRNA1	GGCATACCTGTTATAATCCG
eIF4A2_sgRNA2	GTGGCTCCGCGGATTATAAC
eIF4A2_sgRNA3	GCGCCCGACTGAAAAGACAG

Table S2. Amino acid sequences of recombinant proteins. Cleaved His-tag residues are shown in red, and the TEV cleavage site is indicated by an asterisk. The red-colored peptide is removed during purification and is not present in the protein used for assays.

eIF4A1 WT
MHHHHHHENLYFQ*GSASQDSRSRDNGPDGMEPEGVIESNWNEIVDSFDDMNLSESLLRGIY
AYGFEKPSAIQQRAILPCIKGYDVIAQAQSGTGKTATFAISILQQIELDLKATQALVLAPTRELAQQ
IQKVVMALGDYMGASCHACIGGTNVRAEVQKLQMEAPHIIVGTPGRVFDMLNRRYLSPKYIKM
FVLDEADEMLSRGFKDQIYDIFQKLNSNTQVVLLSATMPSDVLEVTKKFMRDPIRILVKKEELTL
EGIRQFYINVEREEWKLDTLCDLYETLTITQAVIFINTRRKVDWLTEKMHARDFTVSAMHGDMD
QKERDVIMREFRSGSSRVLITTDLLARGIDVQQVSLVINYDLPTNRENYIHRIGRGGRFGRKGVA
INMVTEEDKRTLRDIETFYNTSIEEMPLNVADLI

eIF4A2 WT
MHHHHHHENLYFQ*GSGGSADYNREHGGPEGMDPDGVIESNWNEIVDNFDDMNLKESLLRGI
YAYGFEKPSAIQQRAIIPCIKGYDVIAQAQSGTGKTATFAISILQQLEIEFKETQALVLAPTRELAQ
QIQKVILALGDYMGATCHACIGGTNVRNEMQKLQAEAPHIVVGTPGRVFDMLNRRYLSPKWIK
MFVLDEADEMLSRGFKDQIYEIFQKLNTSIQVVLLSATMPTDVLEVTKKFMRDPIRILVKKEELTL
EGIKQFYINVEREEWKLDTLCDLYETLTITQAVIFLNTRRKVDWLTEKMHARDFTVSALHGDMD
QKERDVIMREFRSGSSRVLITTDLLARGIDVQQVSLVINYDLPTNRENYIHRIGRGGRFGRKGVA
INFVTEEDKRILRDIETFYNTTVEEMPMNVADLI

eIF4A1 D198E
MHHHHHHENLYFQ*GSASQDSRSRDNGPDGMEPEGVIESNWNEIVDSFDDMNLSESLLRGIY
AYGFEKPSAIQQRAILPCIKGYDVIAQAQSGTGKTATFAISILQQIELDLKATQALVLAPTRELAQQ
IQKVVMALGDYMGASCHACIGGTNVRAEVQKLQMEAPHIIVGTPGRVFDMLNRRYLSPKYIKM
FVLDEADEMLSRGFKDQIYEIFQKLNSNTQVVLLSATMPSDVLEVTKKFMRDPIRILVKKEELTLE
GIRQFYINVEREEWKLDTLCDLYETLTITQAVIFINTRRKVDWLTEKMHARDFTVSAMHGDMDQ
KERDVIMREFRSGSSRVLITTDLLARGIDVQQVSLVINYDLPTNRENYIHRIGRGGRFGRKGVAI
NMVTEEDKRTLRDIETFYNTSIEEMPLNVADLI

Table S3. List of antibodies

Target	Supplier	Cat. No.	Utility	Dilution
eIF4A1	MedChemExpress	HY-P80650	Immunofluorescence	1:500
eIF4A1	Abcam	ab31217	Western blot	1:1000
	Abcam	ab31217	CoIP	
eIF4A2	Abcam	ab31218	Immunofluorescence	1:500
eIF4A2	Santa Cruz Biotechnology	SC-137148	Western blot	1:500
	Santa Cruz Biotechnology	SC-137148	CoIP	
GAPDH	Cell Signaling Technology	2118S	Western blot	1:5000
Puromycin	Sigma	MABE343	Western blot	1:1000
Myc	Cell Signaling Technology	18583S	Western blot	1:1000
r-H2AX	Cell Signaling Technology	80312S	Western blot	1:1000
β-actin	Cell Signaling Technology	3700S	Western blot	1:5000
G3BP1	Cell Signaling Technology	61559	Immunofluorescence	1:500
TIAR	Cell Signaling Technology	8509	Immunofluorescence	1:500

Chemical Synthesis and Characterizations

General Experiment Procedure

Unless otherwise noted, all reactions were carried out in oven-dried glassware fitted with rubber septa under a positive pressure of argon. Air- and moisture-sensitive reagents and solvents were transferred using syringe techniques. Reaction mixtures were concentrated by rotary evaporation at temperatures ≤40 °C.

Analytical thin-layer chromatography (TLC) was performed on glass plates pre-coated with silica gel (0.25 mm thickness, 60 Å pore size, 230–400 mesh; Merck KGaA) containing a fluorescent indicator (254 nm). TLC plates were visualized under ultraviolet light and subsequently stained by immersion in either a 10% (w/v) solution of phosphomolybdic acid (PMA) in ethanol or a 2% (w/v) aqueous solution of potassium permanganate, followed by brief heating.

Flash column chromatography was conducted using a Teledyne ISCO CombiFlash EZ Prep system equipped with pre-packed silica gel cartridges (Teledyne ISCO RediSep).

Solvents and reagents

Anhydrous solvents were purchased from Acros Organics. Unless otherwise specified, all chemical reagents were obtained from Sigma-Aldrich, AK Scientific, or Chemscene. The synthesis of rocagloic acid and its enantiomer was carried out by Medicilon Inc. (Shanghai) following procedures reported by John Porco and coworkers.[1] Enantiomer separation was achieved by supercritical fluid chromatography (SFC). The characterization data of the final products matched the previously reported values. Commercial solvents and reagents were used as received without further purification.

Instrumentation and analytical methods

^1H NMR spectra were recorded on a Bruker Avance III HD spectrometer (400 MHz for ^1H) at 23 °C using Bruker TopSpin 3.1 software. Spectra were processed using MestReNova (version 14.1.2). Proton chemical shifts (δ) are reported in parts per million (ppm) relative to residual solvent signals ($CDCl_3$: δ 7.26; DMSO-d6: δ 2.50). Multiplicity is reported using standard abbreviations (s, d, t, q, dd, dt, m, br, app), and data are presented as chemical shift, multiplicity, integration, and coupling constant (J, Hz). High-resolution mass spectra were acquired on a Waters Xevo G2-XS time-of-flight mass spectrometer operated with Waters MassLynx software (version 4.2).

For the condition of LC-MS: An aliquot (1 µL) of the reaction mixture (or the organic phase of a mini-workup mixture) was diluted with 100 µL 1:1 acetonitrile:water. 1 µL of the diluted solution was injected onto a Waters Acquity UPLC BEH C18 1.7 µm column and eluted with a linear gradient of 5–95% acetonitrile/water (+0.1% formic acid) over 3.0 min. Chromatograms were recorded with a UV detector set at 254 nm and a time-of-flight mass spectrometer (Waters Xevo G2-XS). The purity profile of the final product was determined by the same method.

Safety statement

No unexpected or unusually high safety hazards were encountered. All chemical syntheses were performed in ventilated fume hoods operating at a face velocity of 90 fpm.

Synthesis of Roc-PEG11-amine

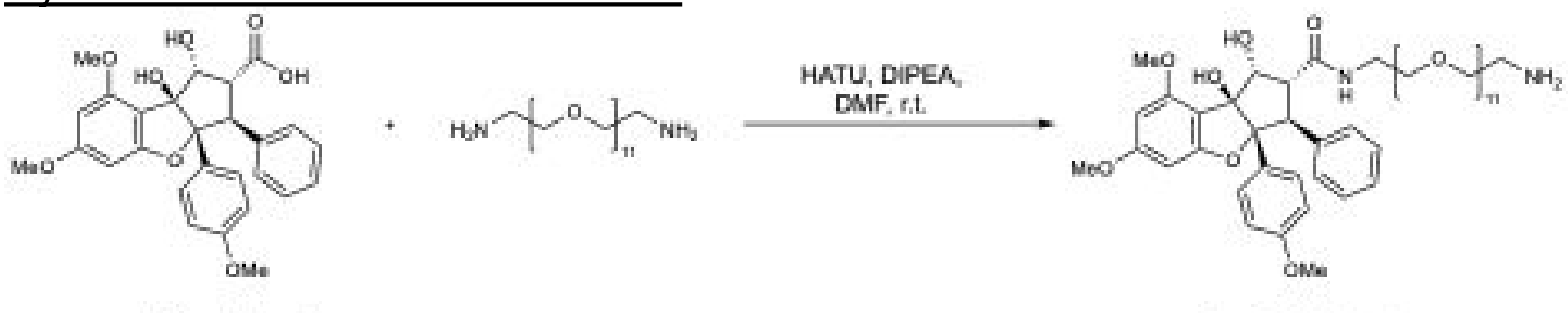

Rocagloic acid Roc-PEG11-amine

Rocagloic acid (50 mg, 0.10 mmol, 1.0 equiv.) and HATU (43.7 mg, 0.11 mmol, 1.1 equiv.) were added to a 20 mL reaction vial, followed by DMF (0.5 ml). DIPEA (55.5 ul, 0.30 mol, 3.0 equiv.) was added dropwise to the reaction. The mixture was stirred for 5 min at room temperature before addition of unprotected PEG11 diamine (227.5 mg, 0.42 mol, 4.2 equiv.). The reaction mixture turned yellow upon addition of the amine and was stirred until complete conversion of the starting material, as monitored by LC-MS. The crude mixture was purified directly by C18 column without aqueous workup. The product was eluted with 70% acetonitrile in water containing 0.1% formic acid (ACN, 0.1% FA). The solvent was removed by lyophilization to afford the product as a white powder (59.4 mg, 0.059 mol, 56% yield).
^{1}H NMR (400 MHz, CD_3CN) 7.28 (t, *J* = 5.7 Hz, 1H), 7.17 – 6.98 (m, 7H), 6.67 – 6.59 (m, 2H), 6.25 (d, *J* = 2.0 Hz, 1H), 6.14 (d, *J* = 2.0 Hz, 1H), 4.63 (d, *J* = 5.3 Hz, 1H), 4.31 (d, *J* = 14.1 Hz, 1H), 3.90 (dd, *J* = 14.1, 5.3 Hz, 1H), 3.85 – 3.48 (m, 55H), 3.48 – 3.36 (m, 2H), 3.32 – 3.24 (m, 2H), 2.97 (t, *J* = 5.2 Hz, 2H). [with an additional proton due to amine protonation by formic acid].
HRMS (m/z): calculated for $C_{51}H_{77}N_2O_{18}$ $[M + H]^+$ 1005.5171, found 1005.5020.

Synthesis of HemiRoc

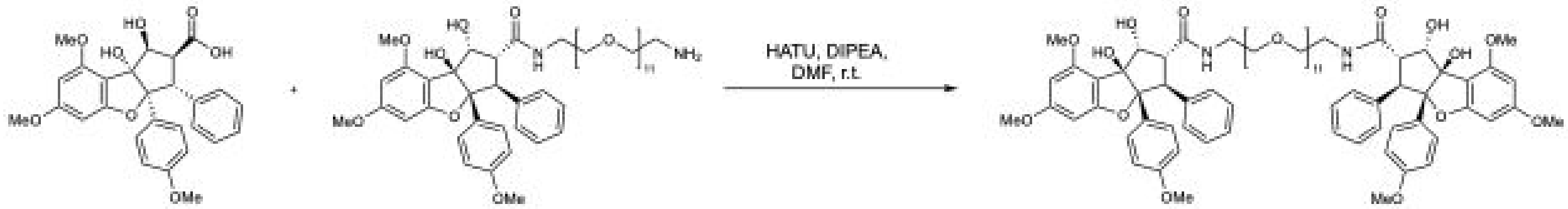

ent-Rocagloic acid HemiRoc

ent-Rocagloic acid (7.2 mg, 0.015 mmol, 1.0 equiv.) and HATU (5.7 mg, 0.015 mmol, 1.0 equiv.) were added to a 4 mL reaction vial, followed by DMF (0.2 ml). DIPEA (8 ul, 0.045 mol, 3.0 equiv.) was added dropwise to the reaction. The mixture was stirred for 5 min at room temperature before addition of Roc-PEG11-amine (11.0 mg, 0.016 mol, 1.1 equiv.). The reaction mixture turned yellow upon addition of the amine and was stirred until complete conversion of the starting material, as monitored by LC-MS. The crude mixture was purified directly by C18 column without aqueous workup. The product was eluted with 95% acetonitrile in water containing 0.1% formic acid (ACN, 0.1% FA). The solvent was removed by lyophilization to afford the product as a white powder (3.1 mg, 0.0021 mol, 14% yield).
^{1}H NMR (400 MHz, DMSO) δ 8.31 (t, *J* = 5.7 Hz, 2H), 7.08 – 6.91 (m, 14H), 6.62 – 6.53 (m, 4H), 6.26 (d, *J* = 1.9 Hz, 2H), 6.10 (d, *J* = 2.0 Hz, 2H), 4.96 (s, 2H), 4.59 – 4.50 (m, 4H), 4.17 (d, *J* = 14.1 Hz, 2H), 3.85 (dd, *J* = 14.1, 5.2 Hz, 2H), 3.77 (s, 6H), 3.72 (s, 6H), 3.59 (d, *J* = 3.9 Hz, 6H), 3.53 – 3.42 (m, 40H), 3.38 – 3.25 (m, 4H), 3.19 – 3.10 (m, 4H).
HRMS (m/z): calculated for $C_{78}H_{101}N_2O_{25}$ $[M + H]^+$ 1465.6693, found 1465.6493.

Synthesis of RocA-PAL

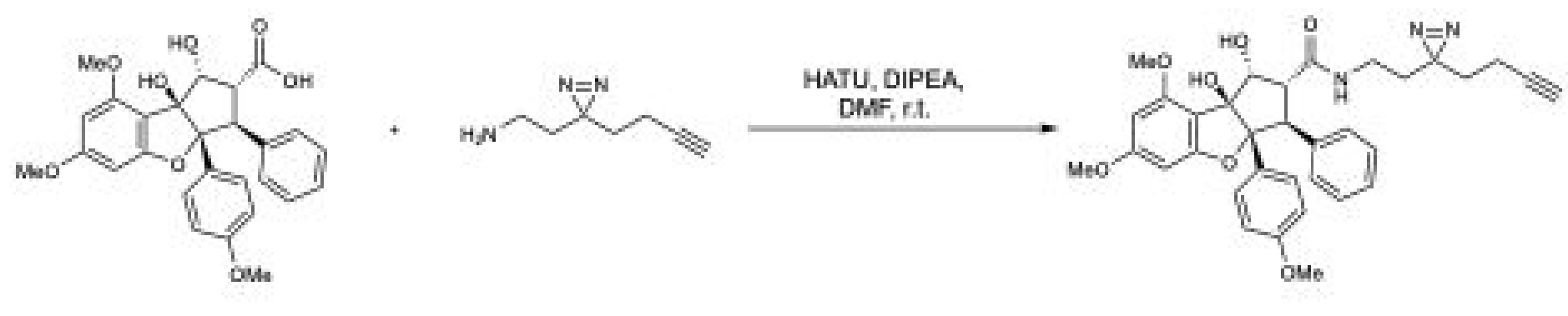

Rocagloic acid RocA-PAL

Rocagloic acid (5.0 mg, 0.01 mmol, 1.0 equiv.) and HATU (5.7 mg, 0.015 mmol, 1.5 equiv.) were added to a 4 mL reaction vial, followed by DMF (0.5 mL). DIPEA (6 ul, 0.030 mmol, 3.0 equiv.) was added dropwise to the reaction. The mixture was stirred for 5 min at room temperature before addition of minimalist diazirine alkyne amine (2.7 mg, 0.02 mmol, 2.0 equiv.). The reaction mixture turned yellow upon addition of the amine and was stirred until complete conversion of the starting material, as monitored by LC-MS. The crude mixture was purified directly by C18 column without aqueous workup. The product was eluted with 95% acetonitrile in water containing 0.1% formic acid (ACN, 0.1% FA). The solvent was removed by lyophilization to afford the product as a white powder (6.0 mg, 0.01 mol, 96% yield).

^{1}H NMR (400 MHz, DMSO) δ 8.22 (t, *J* = 5.6 Hz, 1H), 7.08 – 6.91 (m, 7H), 6.63 – 6.56 (m, 2H), 6.27 (d, *J* = 2.0 Hz, 1H), 6.11 (d, *J* = 2.0 Hz, 1H), 4.97 (s, 1H), 4.56 (dd, *J* = 5.3, 3.4 Hz, 1H), 4.51 (d, *J* = 3.3 Hz, 1H), 4.18 (d, *J* = 14.1 Hz, 1H), 3.81 – 3.75 (m, 4H), 3.73 (s, 3H), 3.60 (s, 3H), 2.88 (p, *J* = 6.8 Hz, 2H), 2.81 (t, *J* = 2.7 Hz, 1H), 1.94 (td, *J* = 7.4, 2.7 Hz, 2H), 1.52 (t, *J* = 7.2 Hz, 2H), 1.45 (td, *J* = 7.1, 3.9 Hz, 2H).

HRMS (m/z): calculated for $C_{34}H_{36}N_3O_7$ $[M + H]^+$ 598.2553, found 598.2604.

Methods

Safety statement

No unexpected or unusually high safety hazards were encountered. All experiments were performed with standard personal protective equipment (PPE). Handling of Biosafety Level 2 materials was performed according to UCSF Office of Environment, Health and Safety standards.

Recombinant protein expression and purification

The DNA sequences encoding human eIF4A1 WT, eIF4A1 D198E, and eIF4A2 were synthesized by Genscript. They were then cloned into the pET21a(+) vector with an N-terminal TEV-cleavable 6xHis tag.

BL21(DE3) chemically competent cells (NEB, C2527H) were transformed with the pET21a(+)-His-TEV-eIF4A expression plasmids and spread onto LB agar plates containing 100 µg/mL ampicillin. Plates were incubated overnight at 37 °C, and a single resulting colony was used to inoculate Terrific Broth supplemented with 100 µg/mL ampicillin. The culture was grown at 37 °C with shaking at 200 rpm until reaching an OD_{600} of approximately 0.6. Expression was initiated by adding 0.5 mM IPTG at 18 °C for overnight induction. Cells were collected by centrifugation at 4,500 × g for 15 min, resuspended in lysis buffer (20 mM HEPES pH 7.5, 500 mM NaCl, 10 mM imidazole, 2 mM DTT), and lysed by sonication. After clarification by centrifugation at 19,000 × g for 1 h, the supernatant was incubated with Co-TALON resin (Clontech; 4 mL slurry per liter culture) for 1 h at 4 °C with gentle rotation. The resin was washed three times with lysis buffer (10 mL each), and bound His-tagged eIF4A proteins were eluted using 20 mM HEPES pH 7.5, 500 mM NaCl, 250 mM imidazole, and 2 mM DTT (15 mL total).

His-tag removal was performed by overnight digestion with His-tagged TEV protease (Macrolab, UC Berkeley) during dialysis against 20 mM HEPES pH 7.5, 200 mM NaCl, and 2 mM DTT at 4 °C. The digest was briefly incubated with HisPur Ni-NTA resin (Thermo Fisher; 1 mL slurry per liter culture) for less than 10 min, and the unbound fraction containing cleaved eIF4A protein was collected. This material was concentrated to ~20 mg/mL using a 10 kDa MWCO centrifugal concentrator (Vivaspin Turbo 15, Sartorius) and further purified by size-exclusion chromatography on a Superdex 200 10/300 GL column (GE Healthcare) equilibrated in SEC buffer (20 mM HEPES pH 7.5, 150 mM NaCl, 1 mM DTT). Peak fractions were pooled, concentrated to ~5 mg/mL, and stored at −78 °C. Typical yields were 5–10 mg purified eIF4A protein per liter of bacterial culture. The mass of the final protein product was verified by LC-MS.

The truncated DDX3X 132-607 protein was prepared according to the reported procedures.[2]

Fluorescence polarization assays

Purified eIF4A proteins were serially diluted on 384-well plates (Corning, 4514). Compounds and 5'FAM-labeled RNA probes (Genscript) were then added to yield final reaction conditions of 50 μM compound, 10 nM 5'FAM RNA, 20 mM HEPES (pH 7.5), 150 mM NaCl, 1 mM DTT, 1 mM AMP-PNP, and 1 mM $MgCl_2$ in a total volume of 20 μL. Reactions were incubated at room temperature for 30 min, after which fluorescence polarization was measured on a Spark multimode plate reader (Tecan) at room temperature. mP values were plotted against eIF4A concentrations and binding curve was fit using GraphPad Prism 10.

Native gel electrophoretic mobility-shift assays

The eIF4A protein (10 μM) and 5'FAM-[AG] RNA probe (100 nM) were assembled with the compound (50 μM) in reaction buffer (20 mM HEPES pH 7.5, 150 mM NaCl, 1 mM DTT, 1 mM AMP-PNP, and 1 mM $MgCl_2$). After incubation at room temperature for 30 min, 2× native loading buffer (Invitrogen, LC2673) was added, and the mixture was loaded onto an 8–16% native gel (Invitrogen, XP08160BOX). Electrophoresis was performed in Tri-Glycine native running buffer (Invitrogen, LC2672) at 200 V for 40 min. The gel was first scanned for fluorescein signal, followed by Coomassie Blue staining and a second scan to visualize total protein.

In vitro transcription

The different 5'-UTR sequences were inserted into luciferase reporter plasmid pGL4.10[luc2] by Genwiz. AGA 5'-UTR: 5′-AAGCTTGAA(AGAGAGCAACAA)6AGAGAGCACCAAGCTTGGCAATCCGGTACTGTTGGTAAAGCCACC-3′. CAA 5'-UTR: 5′-AAGCTTGAA(CAACAACAACAA)6CAACAACACCAAGCTTGGCAATCCGGTACTGTTGGTAAAGCCACC-3′. CCG 5'-UTR: 5′-AAGCTTGAA(CCGCCGCAACAA)6CCGCCGCACCAAGCTTGGCAATCCGGTACTGTTGGTAAAGCCACC-3′. The resulting plasmids constructs contained a T7 promoter followed by the customized 5'-UTR sequence and the luciferase coding sequence.

The DNA templates were linearized by digestion with Bam HI (NEB) and purified with the QIAquick PCR Purification kit (QIAGEN) according to manufacturer's instructions. Templates were in vitro transcribed using the mMessage mMachine T7 ULTRA transcription kit and purified using the MEGAclear kit (Ambion) according to manufacturer's instructions.

In vitro translation assays

The experiments were conducted based on Rabbit Reticulocyte Lysate, Nuclease-Treated (Promega) following manufacture protocol. The in vitro transcribed mRNA reporters were added to the Rabbit Reticulocyte Lysate to a final concentration of 10 ng/µl and incubated for 90 min at 30°C prior to the measurement of luciferase activities using ONE-Glo™ Luciferase Assay System (Promega).

Cell culture

K562, 22Rv1, HEL, KELLY cells (UCSF Cell Culture Facility) were cultured in RPMI 1640 (Gibco, 11995073) supplemented with 4 mM L-glutamine, 1 mM sodium pyruvate, and 10% heat-inactivated FBS (Axenia Biologix). HEK293T cells (UCSF Cell Culture Facility) were maintained in DMEM (Gibco, 11995073) with the same supplements. The K562 CRISPRi (K562i) line was generated as described previously.[3] G-401 cells (UCSF Cell Culture Facility) were cultured in McCoy's 5a Medium Modified and 10% heat-inactivated FBS (Axenia Biologix).
All the cells were grown at 37 °C with 5% CO_2 under standard stationary culture conditions unless noted otherwise. All cell lines tested negative for mycoplasma using the MycoAlert Mycoplasma Detection Kit (Lonza).

DNA transfections and lentivirus production

HEK293T cells were transfected with sgRNA expression vectors and standard packaging vectors (Cellecta, catalog no. CPCP-K2A) using Lipofectamine 3000 Transfection Reagent (Invitrogen, catalog no. L3000015). Lentiviral supernatant was collected 2 days following transfection, filtered through sterile 0.45 µm polyvinylidene difluoride filters (Millipore), and used immediately or stored at -80 °C.

CRISPRi screening

K562 CRISPRi cells were grown at 37 °C and 5% CO_2 in 1 L Nalgene Disposable Erlenmeyer flasks with Vented Closure (Thermo Scientific 4112-1000) in a shaking culture (1300 rpm) in a Multitron Incubator (Infors HT). Cells were cultured in RPMI (Gibco) supplemented with 10% fetal bovine serum (FBS) (Avantor Seradigm), penicillin (100 U/mL, Gibco), streptomycin (100 µg/mL, Gibco), glutamine (29.2 µg/mL, Gibco 10378016), and 0.1% Pluronic F-68 (Gibco, Thermo 24040032). Cells were transduced with the 5-sgRNA/gene human CRISPRi v2 library (hCRISPRi-v2) with 8 µg/mL polybrene. Library virus was titered to maximally transduce cells while maintaining a multiplicity of infection (MOI) less than 1. Transduction efficiency and cell growth and viability was monitored daily throughout the experiment by flow cytometry (Attune II, Thermo

Fisher). sgRNA+ cells were selected with doses of 1 µg/mL puromycin at 48 and 72 hrs post-transduction until the population was 80-90% sgRNA positive, then cells were allowed to recover in fresh media for 24 hrs before compound treatment. T0 samples were collected before treatment at approximately 750-fold coverage of the library (750 million cells) and the rest of the cells were split into 4 treatment arms with 2 biological replicates per arm: DMSO, 10 nM RocA, 5 nM BisRoc. Cells were cultured at a minimum of 750- to 1000-fold library coverage and continued to be monitored daily by flow cytometry. Dilutions were made daily as necessary with media supplemented with treatments at the indicated concentration to maintain selective pressure until the end of the experiment on Day 11 of compound treatment. The only exception was the BisRoc treatment arms, in which compound was removed after Day 7 of treatment in order to decrease selective pressure to maintain adequate library coverage. Samples were harvested on Day 11 at a minimum of 1000-fold coverage (or 750-fold coverage for BisRoc arms). NucleoSpin Blood XL kits (Macherey-Nagel, Takara Bio 740950.10) were used to extract genomic DNA (gDNA) from all samples and sgRNA protospacers were amplified directly from this with Ti Taq polymerase (Takara Bio 639209), analyzed on an Agilent Tapestation on a High Sensitivity DNA D1000 ScreenTape (Agilent 5067-5584 and 5067-5585), and then sequenced on an Illumina HiSeq 4000 through the UCSF Center for Advanced Technology (UCSF CAT).

Cloning of single sgRNA expression vectors

sgRNA protospacers targeting individual genes or a negative control were chosen from the library and cloned into the vector pCRISPRia-v2 (Addgene 84832) as previously described.[3] Complementary oligonucleotides (Elim Bio) were annealed by mixing in Nuclease-Free Duplex Buffer (Integrated DNA Technologies) and heated at 95 °C for 5 min, then cooled at 22 °C for 1 hr. The vector pCRISPRia-v2 was digested by BstXI and BlpI (New England Biolabs) and gel purified, then ligated to the annealed duplex with fresh T4 DNA Ligase (New England Biolabs M0202S). Ligated plasmids were transformed into Stellar Competent Cells (Takara Bio 636763) and grown overnight. Individual colonies were grown overnight in 3 mL liquid cultures, plasmids were purified with the Qiagen Spin Miniprep Kit, and sequenced by standard Sanger sequencing (Elim Bio).

Stable cell line generation

K562 CRISPRi cells (2×10^5) were seeded in 1 mL media in 24-well plates, grown overnight, and treated with lentivirus containing individual sgRNA vectors and 8 µg/mL polybrene (Sigma, TR-1003-G). Cells were treated with 2 µg/mL puromycin (Invivogen, ANT-PR-801) beginning 48

hours post-transduction and puromycin was maintained for 10 days until populations were pure by flow cytometry (CytoFLEX, Beckman Coulter). Knockdowns were checked by immunoblotting over the course of subsequent experiments.

Cell viability assays

1000 cells were seeded in 100 µL media in 96 well plates and grown overnight. Cells were treated in triplicate with compounds with 0.5% DMSO. Compounds were added by Tecan D300e digital dispenser. Plates were incubated at 37 °C with 5% CO_2 for 72 h following treatment. 100 µL of a solution of 1:4 CellTiter-Glo (Promega, G7572) to PBS was added per well and plates were shaken for 10 min to mix before luminescence was read out on a Tecan Spark plate reader. The K562i IFITM1-3 sg1, IFITM1-3 sg2, TriNegCtrl cell lines are from previous work in our lab.[4]

Cancer cell line panel screening

High-throughput drug screening was performed as previously described.[5] 300 cancer cell lines were tested for mycoplasma and grown in medium supplemented with penicillin/streptomycin and 10% FBS in a humidified atmosphere at 37°C with 5% CO_2. All cell lines were grown in RPMI or DMEM/F12 medium to facilitate high-throughput screening and to minimize potential effects of different media on drug sensitivity. Cells were seeded in 384-well plates at varying density to ensure optimal growth of each cell line over the course of the experiment. Cells were treated with vehicle (DMSO) or compound stocks dissolved in DMSO with a PerkinElmer JANUS workstation the day following seeding. Compounds were given in duplicate at 9 doses with a 3-fold dilution factor. The highest dose of RocA was 1 µM and the highest dose of BisRoc was 3 µM. A Day 0 readout was done for growth-rate normalization. Cell viability was determined by CellTiter-Glo after five days of compound treatment and compound-treated wells were normalized to DMSO-treated wells.

Correlation analysis between cell viability and gene expression

Dose-dependent inhibition of 300 cancer cell lines by RocA and BisRoc was determined first, where dose-response curves were fitted to a four-parameter log-logistic model using SciPy scipy.optimize.curve_fit to estimate IC_{50} values. Correlations of drug sensitivity were determined using the cellpanelr web-app[6] (https://dwassarman.shinyapps.io/cellpanelr/) with gene expression and proteomics data obtained from the DepMap (22Q1 release) and plotted in GraphPad Prism 10.

SDS–PAGE and immunoblotting

K562 cells (2 mL medium, 10^6/mL) were treated with compounds with a final DMSO concentration of 0.5%. At the end of the treatment period, cells were placed on ice. Unless otherwise indicated, cells were pelleted by centrifugation (500g, 5 min), washed once with 1 ml ice-cold PBS, and pelleted again. Unless otherwise specified, cell pellets were lysed on ice for 10 min in RIPA buffer supplemented with protease and phosphatase inhibitors (cOmplete Mini and PhosSTOP, Roche). Protein concentrations were determined using a BCA assay (Thermo Fisher, 23225) and adjusted to 2 mg/ml, or to the highest achievable concentration, with additional RIPA buffer. Lysates were mixed with 4x SDS loading dye (BioRad, 1610747) and denatured at 95 °C for 5 min for immunoblotting.

Unless otherwise specified, SDS–polyacrylamide gel electrophoresis was performed using 4-12% Bis-Tris gels (Novex, Invitrogen) in MES running buffer at 200 V for 50 min. Proteins were transferred onto nitrocellulose membranes using iBlot 3 Transfer Stacks (Invitrogen, IB33002X3). Membranes were blocked in 5% bovine serum albumin (BSA) in Tris-buffered saline with Tween-20 (TBST) for 1 h at 23 °C. Primary antibodies were diluted in 5% BSA–TBST and incubated with membranes at 4 °C for overnight. Membranes were washed three times with TBST (5 min per wash) and incubated with IRDye-conjugated secondary antibodies (goat anti-rabbit IgG-IRDye 800 or goat anti-mouse IgG-IRDye 680; Li-COR) diluted in 5% BSA–TBST for 1 h at 23 °C. Following three additional TBST washes (5 min each), membranes were imaged using a Bio-Rad ChemiDoc Imager.

Puromycin incorporation assays

K562 cells (2 mL medium, 10^6cells/mL) were incubated with the drug for 5 hours, followed by the addition of 5 µg/ml puromycin (InvivoGen). The cells were incubated for an additional 60 min before cell harvest and lysis. The western blot was incubated with anti-puromycin to detect the extent of puromycin incorporation.

qRT-PCR analysis

RNA from treated cells was isolated using the RNeasy Plus Mini Kit (QIAGEN) according to manufacturer instructions. Reverse transcription was performed using the QuantiTect Reverse Transcription Kit (QIAGEN) according to manufacturer instructions. The reverse transcription products were evaluated by qRT-PCR using the Maxima SYBR Green qPCR Master Mix (Life Technologies) or SsoAdvanced Universal SYBR Green Supermix (Bio-Rad) on a Bio-Rad CFX Touch Real-Time PCR system according to manufacturer instructions. GAPDH served as a

reference gene. All samples were evaluated using the ΔΔCq method under the gene expression tab in the Bio-Rad CFX Maestro for Mac 1.1 software. Primer sequences are as follows: GAPDH forward: 5′-AAT CCC ATC ACC ATC TTC CA-3′; GAPDH reverse: 5′-TGG ACT CCA CGA CGT ACT CA-3′; MYC forward: 5’-CAC CAG CAG CGA CTC TGA-3’; MYC reverse: 5’-GAT CCA GAC TCT GAC CTT TTG C-3’.

Puromycin incorporation proteomics

Similarly, K562 cells (10 mL medium, 10^6 cells/mL) were incubated with the DMSO, RocA (100 nM), or BisRoc (100 nM) for 5 h (n=3), followed by the addition of 10 µM O-Propargyl-Puromycin (OPP; BroadPharm, BP-50103). Cells were incubated for an additional 60 min prior to cell harvest and lysis in 0.1% NP40, 100 mM HEPES, 150 mM NaCl pH 7.5 with 1x Halt Protease and Phosphatase Inhibitor Cocktails (Fisher, 78447). Cell lysates were clarified by centrifugation (13,000g, 15 min, 4 °C), and protein concentrations were determined using a BCA assay and normalized to 2 mg/mL.

2 mg cell lysates were subjected to click chemistry by conjugation with 0.1 mM biotin-azide (Sigma, 900912-50MG) in the presence of 1 mM $CuSO_4$, 0.1 mM TBTA (Vector, CCT-1061), and 1 mM TCEP (GOLDBIO). The reaction was carried out at room temperature for 90 min, after which ice-cold acetone was added, and samples were kept at -20 °C to allow complete protein precipitation. Proteins were pelleted by centrifugation at 4,300 g for 15 min and washed once with cold methanol. The resulting protein pellet was resuspended in 250 µL 4% SDS in PBS containing 10 mM DTT to ensure complete solubilization, then diluted to a volume of 5 mL with PBS. 100 µL equilibrated NeutrAvidin beads (Fisher, PI29200) were added to the samples. The samples were rotated end-over-end for 2 h at room temperature. Beads were collected by centrifugation, washed once with 0.2 % SDS in PBS, followed by three washes with PBS.

Beads were transferred to protein LoBind tubes (Fisher, 13-698-794) and incubated with 400 µL of 5 mM DTT in 6 M urea/PBS at 56 °C for 30 min. Proteins were then alkylated with 42 µL of 200 mM IAA (Sigma, I6125) for 30 min in the dark. On-bead digestion was performed using 2 µg trypsin (Promega, VA9000) in 100 µL 100 mM TEAB (Sigma, T7408) at 37 ^{o}C overnight. The supernatant was collected, and beads were washed twice with 50 µL 100 mM TEAB. All fractions were combined to yield 200 µL of digested peptide solution.

0.8 mg TMT10plex Isobaric Label Reagents (Fisher, 90110) dissolved in 90 µL acetonitrile was added to the peptide solution and incubated for 1 h at room temperature. The reaction was quenched with 12 µL 5% hydroxylamine (Sigma, 467804), followed by the addition of 8 µL formic acid. The labeled peptides were pooled, dried by SpeedVac, and desalted using C18 pipette tips

(Fisher, PI87784). Peptides were eluted with three rounds of 100 µL 80% acetonitrile in water containing 0.1% formic acid and dried under vacuum.

LC-MS/MS acquisition and data analysis of TMT-labeled samples

TMT10plex samples were reconstituted in 50 µL of 5% ACN and 0.1% FA in water and analyzed on an Orbitrap Eclipse Tribrid mass spectrometer (Thermo Fisher Scientific) coupled to an Ultimate 3000 RSLCnano system. Mobile phase A consisted of 0.1% formic acid in water, and mobile phase B consisted of 95% acetonitrile with 0.1% formic acid. For each injection, 5 µL of sample was loaded. Peptides were separated on an EASY-Spray C18 column (3 µm, 75 µm × 15 cm; Thermo Fisher Scientific, Cat. No. ES800) at a flow rate of 0.3 µL min^{-1}. Samples were loaded at 4% B for 20 min, followed by a gradient of 4–10.5% B over 2 min, 10.5–45% B over 73 min, 45–84% B over 2 min, holding at 84% B for 3 min, ramping from 84% to 4% B over 2 min, and re-equilibrating at 4% B for 8 min.

TMT reporter ion quantification was performed using real-time search (RTS)-MS3 methods. MS1 spectra were acquired at a resolution of 120 K with m/z scan range of 375-1800, RF lens of 30%, a maximum ion injection time of 50 ms, charge states of 2-6, and a 2scan30s dynamic exclusion time. MS2 spectra were acquired via HCD at a stepped collision energy of 33 ± 5%, in the orbitrap with an isolation width of 0.7 m/z for the TMT 6-plex sample (0.5 m/z for the TMT 11-plex sample), a resolution of 30 K, m/z scan range of 110-3000 and a maximum ion injection time of 75 ms. In RTS-MS3, real-time database searching was performed using the Comet search engine against the reviewed human Swiss-Prot FASTA database with trypsin specified as the digestion enzyme. Methionine oxidation and probe modifications on tyrosine and lysine were set as variable modifications, while carbamidomethylation of cysteine and TMT modifications on N-terminal amines and lysine residues were specified as static modifications. For MS3 acquisition, synchronous precursor selection (SPS) of the top 10 fragment ions was performed, with a maximum injection time of 105 ms. MS3 spectra were acquired in the Orbitrap at a resolution of 50,000 using HCD with a collision energy of 65%.

Peptides were searched against the SwissProt Homo sapiens reference proteome (2021.06.20) using MaxQuant (v.2.0.3.1, https://www.maxquant.org/) with variable methionine oxidation and protein N-terminal acetylation, and fixed cysteine carbamidomethylation. Quantification was performed with reporter ions in MS3. Proteomic statistical analyses were completed using the R programming language, version 4.5.0. Proteins annotated as reverse hits or contaminants were removed, and zero/negative intensities were treated as missing values. For OPP proteomics, Pearson correlation between RocA- and BisRoc-induced log2 fold changes was computed. For

photoaffinity-based proteomic profiling/MS analysis, Log2 fold changes and p values were calculated in R using Student's t-tests (two-tailed, two-sample, equal variance), and final plots were generated using GraphPad Prism 10.

Washout assays

K562 cells (15 mL medium, 10^6 cells/mL) were incubated with compounds at 1 μM for 2 h. Compounds were removed by centrifugation at 500 g for 5 min, and cells were resuspended in an equal volume of fresh medium and incubated at 37 °C for 5 min prior to pelleting. This wash step was repeated twice. Cells were then reconstituted in 15 mL fresh medium and aliquoted into separate wells (2 mL each) and harvested at the indicated time points. Cell pellets were lysed and analyzed by western blot.

To analyze the effects on global proteomics, 5 μg/mL puromycin was added at indicated time points and incubated for an additional 1 h before cell harvest.

Photoaffinity-based proteomic profiling

K562 cells (10 mL PBS, 10^6 cells/mL) were treated with the 5 μM RocA-PAL in the presence of DMSO, RocA (50 μM), or BisRoc (50 μM) at 37 °C for 1 h (n=3). Cells were transferred to 4 °C cold room and irradiated with 365 nm UV light (Analytikjena, UVL-225D) for 10 min. Cells were pelleted by centrifugation at 500 g for 5 min, washed once with PBS, and lysed in 1 mL of 0.1% NP40, 100 mM HEPES, 150 mM NaCl pH 7.5 with 1x Halt Protease and Phosphatase Inhibitor Cocktails (Fisher, 78447).

Subsequent sample preparation, enrichment, digestion, TMT labeling, and desalting were performed as described above for puromycin-incorporation proteomics.

For gel-based fluorescent proteomic profiling, cell lysates (24 μg) were subjected to click chemistry by conjugation with 0.025 mM TAMRA-azide (BroadPharm, BP-40173) in the presence of 1 mM $CuSO_4$, 0.1 mM TBTA (Vector, CCT-1061), and 1 mM TCEP (GOLDBIO). The reaction was carried out at room temperature for 90 min, after which SDS loading buffer (BioRad, 1610747) was added and samples were incubated at room temperature for 30 min. The samples were resolved by SDS-PAGE, scanned for TAMRA fluorescence, and subsequently stained with Coomassie blue.

Cellular thermal shift assays (CETSA)

K562 cells (10 mL medium, 10^6 cells/mL) were treated with DMSO, RocA (10 μM), or BisRoc (10 μM) for 1 h. Then cells were harvested, washed once with cold PBS, and reconstituted to 0.7 mL

ice-cold PBS, and divided into 13 aliquots (50 µL each). Aliquots were heated at indicated temperatures for 3 min and immediately flash-frozen in liquid nitrogen. Cells were lysed by three freeze-thaw cycles (liquid nitrogen freezing followed by thawing at room temperature). Lysates were clarified by centrifugation (13,000 x g, 10 min, 4 °C), and 15 µL of the supernatant was mixed with 5 µL SDS loading buffer, resolved by SDS-PAGE, and analyzed by western blot. Relative signal intensities of eIF4A1 and eIF4A2 were normalized to GAPDH, further normalized to the lowest temperature, plotted against temperatures, and fitted using GraphPad Prism 10.

Co-immunoprecipitation (CoIP)

K562 cells (10 mL medium, 10^6 cells/mL) were treated with DMSO, RocA, BisRoc, or HemiRoc at the indicated concentrations for 6 h. Cells were pelleted by centrifugation (500g, 5 min, 4 °C), washed once with ice-cold PBS, and lysed in IP lysis buffer (Thermo Fisher, 87788) supplemented with 6 mM $MgCl_2$ and 1× Halt protease and phosphatase inhibitor cocktails (Thermo Fisher, 78447). Lysates were clarified by centrifugation (13,000g, 15 min, 4 °C), and protein concentrations were determined by BCA assay and normalized to 1 mg/mL with IP lysis buffer.

Normalized lysates (400 µg) were incubated overnight at 4 °C with 2.5 µg anti-eIF4A1 (Abcam, ab31217), anti-eIF4A2 (Santa Cruz Biotechnology, SC-137148), or IgG control antibodies (Cell Signaling Technology, 3900S or 5415S). Where indicated, RNase A (10 µg/mL; NEB, T3018L) was added during the incubation. Protein A/G magnetic beads (25 µL bead slurry; Thermo Fisher, 88803) were equilibrated, added to the samples, and incubated at room temperature for 1 h. Beads were collected on a magnetic rack and washed twice with IP lysis buffer and once with water.

For western blot analysis, beads were resuspended in 100 µL 1× SDS loading buffer and boiled at 95 °C for 10 min, after which eluates were collected using a magnetic rack.

For proteomics analysis, cells were treated with compounds at a final concentration of 100 nM. Anti-eIF4A1 antibody or IgG control was used as the immunoprecipitation bait. Following the procedures described above, beads were incubated with 50 µL 6 M urea/PBS at 56 °C for 30 min, diluted to 100 µL with 100 mM TEAB, reduced with 7.5 µL 200 mM TCEP at 56 °C for 60 min, and alkylated with 7.5 µL 375 mM iodoacetamide in 200 mM TEAB at 37 °C for 30 min in the dark. 3 µg trypsin in 100 µL 100 mM TEAB was added to the mixture and incubated at 37 °C overnight. Digestion was quenched by addition of 5 µL TFA. Peptides were collected on magnetic rack. The remaining beads were washed twice with 50 µL 100 mM TEAB. The three fractions were combined and desalted using C18 pipette tips (Thermo Fisher, PI87784), eluted with three rounds of 100 µL 80% acetonitrile in water containing 0.1% formic acid, and dried under vacuum.

LC-MS/MS acquisition and data analysis of label-free samples

Label-free samples were reconstituted in 50 μL of 5% ACN and 0.1% FA in water and analyzed on an Orbitrap Eclipse Tribrid mass spectrometer (Thermo Fisher Scientific) coupled to an Ultimate 3000 RSLCnano system. Mobile phase A consisted of 0.1% formic acid in water, and mobile phase B consisted of 95% acetonitrile with 0.1% formic acid. For each injection, 5 μL of sample was loaded.

Peptides were separated on an EASY-Spray C18 column (3 μm, 75 μm × 15 cm; Thermo Fisher Scientific, Cat. No. ES800) at a flow rate of 0.3 μL min^{-1}. Samples were loaded at 2% B for 12 min, followed by a gradient of 2–31.6% B over 72 min, 31.6–52.6% B over 2 min, ramping from 52.6% to 2% B over 2 min, and re-equilibrating at 2% B for 7 min.

MS1 spectra were acquired at a resolution of 120 K with m/z scan range of 375-1500, RF lens of 30%, a maximum ion injection time of 50 ms, charge states of 2-7, and a 30s dynamic exclusion time. MS2 spectra were acquired via HCD at a stepped collision energy of 30%, in the orbitrap with an isolation width of 1.6 m/z for the label free sample, a resolution of 30 K, auto m/z scan range and a maximum ion injection time of 100 ms.

Peptides were searched according to the process mentioned above. Label-free quantification (LFQ) intensities were processed in R. Raw LFQ values were log-transformed as log2(LFQ + 1), and per-replicate enrichment scores (R1–R3) were calculated as the difference between target and matched control channels for DMSO, RocA, and BisRoc. Enrichment matrices were z-score scaled across samples on a per-protein basis. Hierarchical clustering was performed on both rows and columns using Euclidean distance and complete linkage, and heatmaps were generated using pheatmap.

For focused visualization, the top 200 proteins were selected based on mean BisRoc enrichment across replicates and clustered as above. The resulting row dendrogram was partitioned into eight clusters, and the cluster 1 and 2 were subjected to cellular-component Gene Ontology analysis using STRING (https://string-db.org/).

Immunofluorescence microscopy

22Rv1 cells (5,000 cells per well) were seeded into 96-well plates (Cellvis P96-1-N, glass thickness 0.13–0.16 mm) and incubated overnight at 37 °C. Cells were treated with compounds or DMSO as a vehicle control for 24 h. Following treatment, cells were fixed with 3.7% paraformaldehyde in PBS and permeabilized with 0.1% Triton X-100 in PBS. After removal of the permeabilization solution, cells were washed with PBS-T and blocked with 100 μL of 2% BSA in

PBS-T for 1 h at room temperature. Cells were then stained with DAPI (Sigma-Aldrich, D9542-10MG) to visualize DNA and either anti-eIF4A1 antibody (MedChemExpress, HY-P80650) or anti-eIF4A2 antibody (Abcam, ab31218), anti-G3BP1 antibody (Cell Signaling Technology, 61559), anti-TIAR antibody (Cell Signaling Technology, 8509) overnight at 4 °C. Images were acquired using a CSU-W1 Spinning Disk/High Speed Widefield microscopy equipped with a Plan Apo λ 40x/0.95.

For automated image analysis, the percentage of G3BP1 foci positive cells was determined using CellProfiler software.[7] Data are presented as mean ± SD (n = 3).

Supplementary Reference

1. Gerard, B.; Jones, G.; Porco, J. A. A Biomimetic Approach to the Rocaglamides Employing Photogeneration of Oxidopyryliums Derived from 3-Hydroxyflavones. *Journal of the American Chemical Society* **2004**, *126* (42), 13620–13621. DOI: 10.1021/ja044798o.
2. Floor, S. N.; Barkovich, K. J.; Condon, K. J.; Shokat, K. M.; Doudna, J. A. Analog sensitive chemical inhibition of the DEAD-box protein DDX3. *Protein Sci* **2016**, *25* (3), 638–649. DOI: 10.1002/pro.2857 From NLM.
3. Horlbeck, M. A.; Gilbert, L. A.; Villalta, J. E.; Adamson, B.; Pak, R. A.; Chen, Y.; Fields, A. P.; Park, C. Y.; Corn, J. E.; Kampmann, M.; et al. Compact and highly active next-generation libraries for CRISPR-mediated gene repression and activation. *eLife* **2016**, *5*, e19760. DOI: 10.7554/eLife.19760.
4. Lou, K.; Wassarman, D. R.; Yang, T.; Paung, Y.; Zhang, Z.; O'Loughlin, T. A.; Moore, M. K.; Egan, R. K.; Greninger, P.; Benes, C. H.; et al. IFITM proteins assist cellular uptake of diverse linked chemotypes. *Science* **2022**, *378* (6624), 1097–1104. DOI: 10.1126/science.abl5829 (accessed 2026/01/06).
5. Garnett, M. J.; Edelman, E. J.; Heidorn, S. J.; Greenman, C. D.; Dastur, A.; Lau, K. W.; Greninger, P.; Thompson, I. R.; Luo, X.; Soares, J.; et al. Systematic identification of genomic markers of drug sensitivity in cancer cells. *Nature* **2012**, *483* (7391), 570–575. DOI: 10.1038/nature11005.
6. Wassarman, D. R.; Wu, T.; Shokat, K. M. Cellpanelr: identify predictive biomarkers from cell line panel response data. *bioRxiv* **2022**, 2022.2011.2002.514913. DOI: 10.1101/2022.11.02.514913.
7. Stirling, D. R.; Swain-Bowden, M. J.; Lucas, A. M.; Carpenter, A. E.; Cimini, B. A.; Goodman, A. CellProfiler 4: improvements in speed, utility and usability. *BMC Bioinformatics* **2021**, *22* (1), 433. DOI: 10.1186/s12859-021-04344-9.

^{1}H NMR and ^{1}H-^{1}H COSY of Roc-PEG11-amine

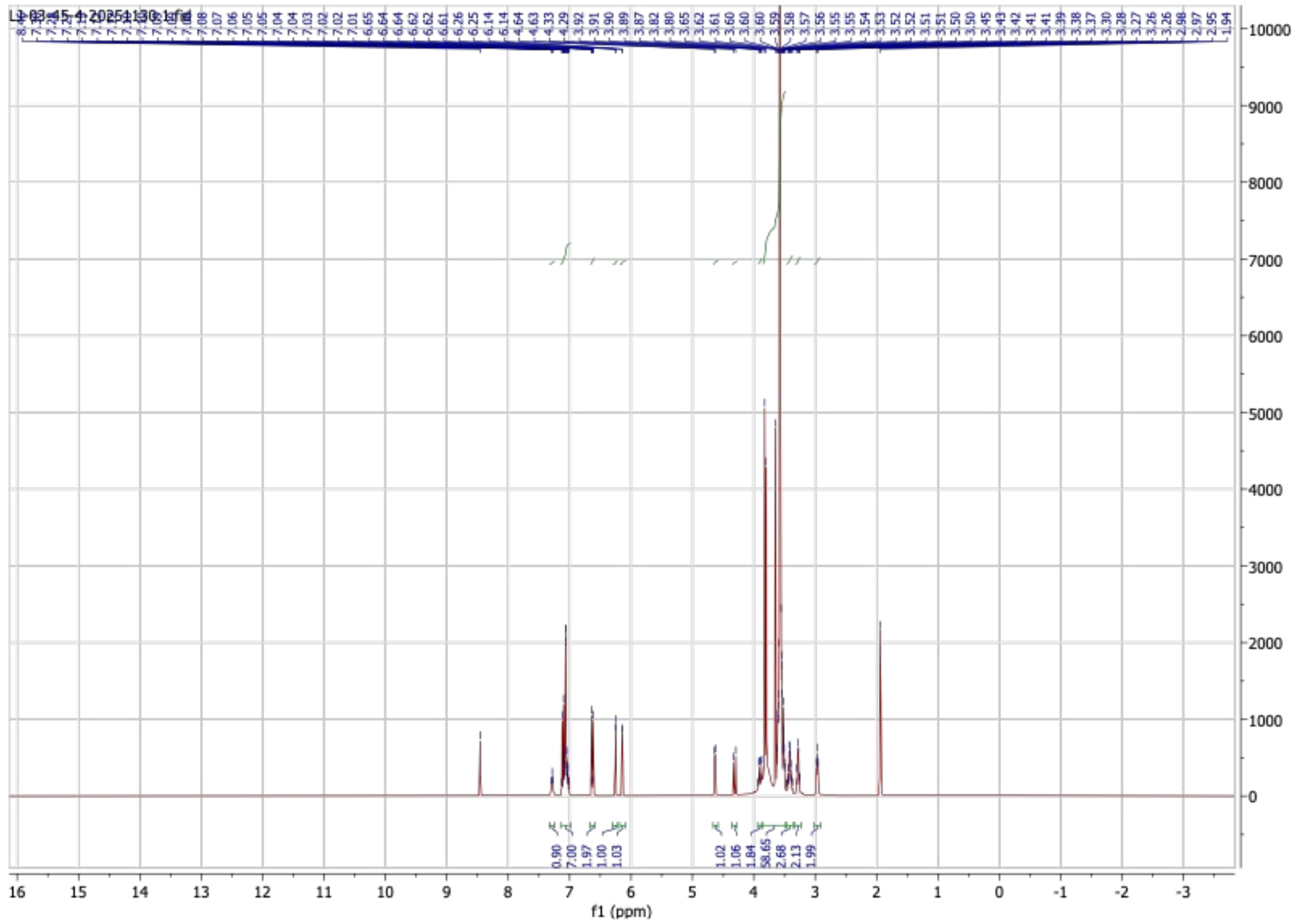

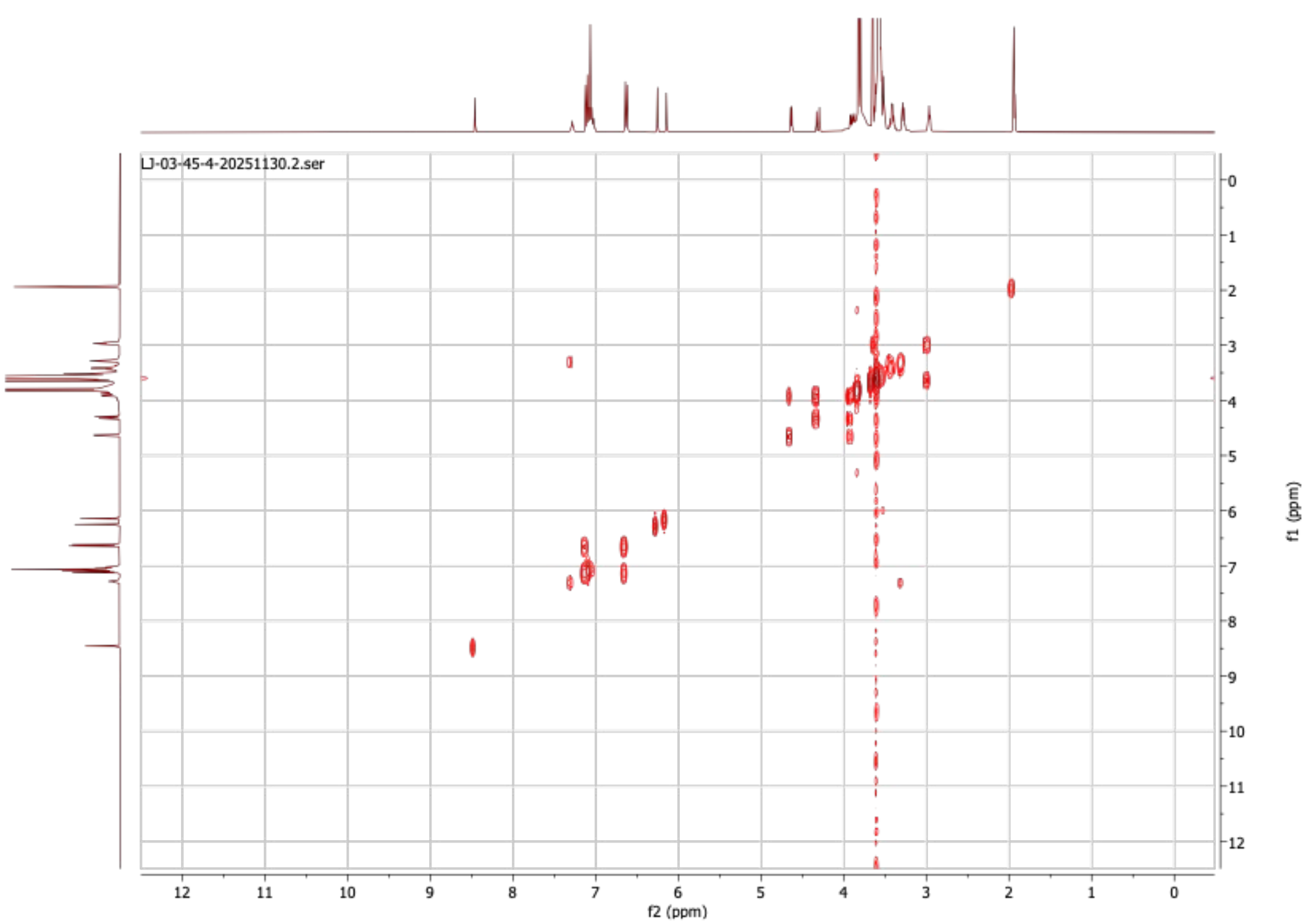

^{1}H NMR and ^{1}H-^{1}H COSY of HemiRoc

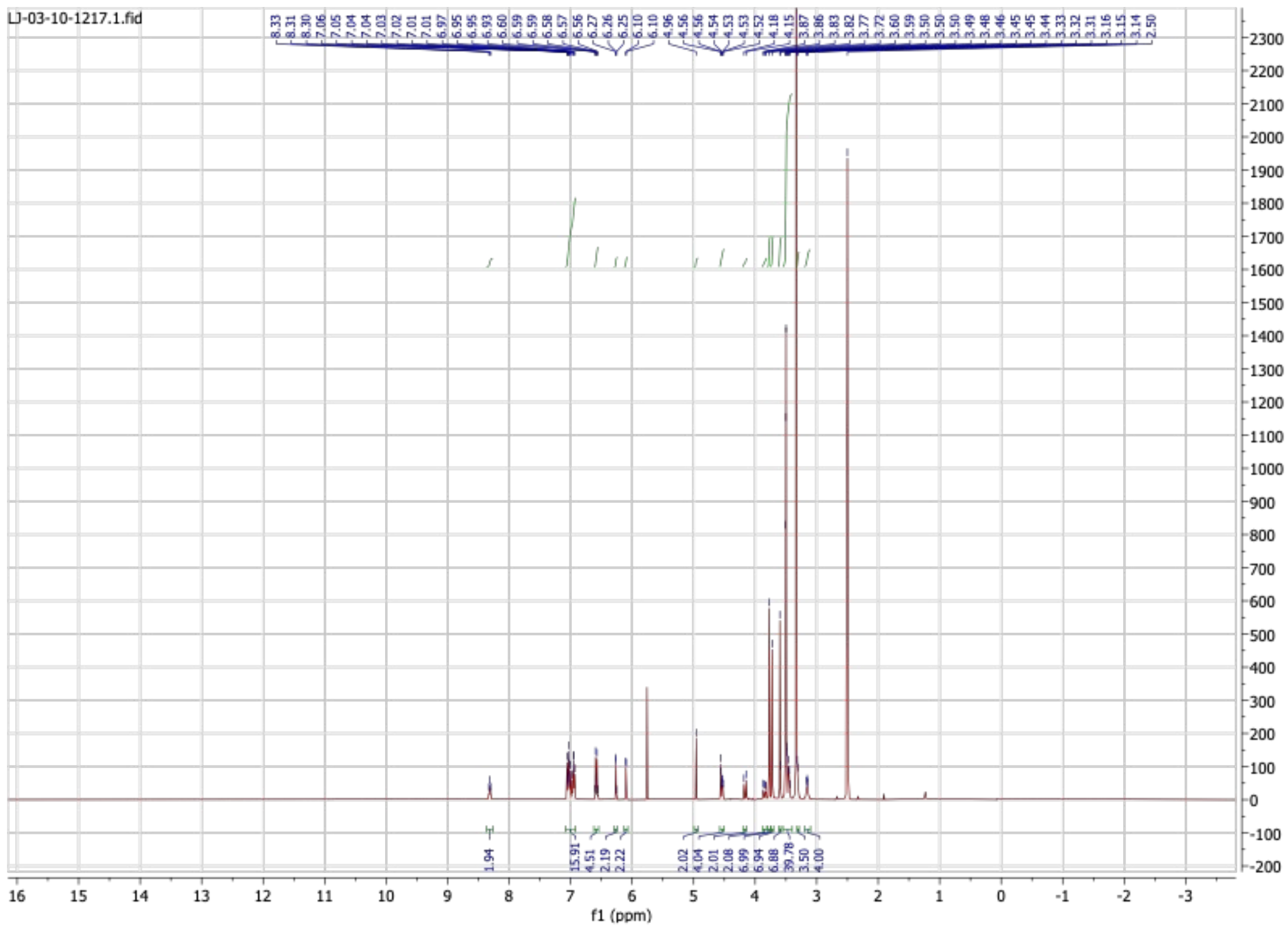

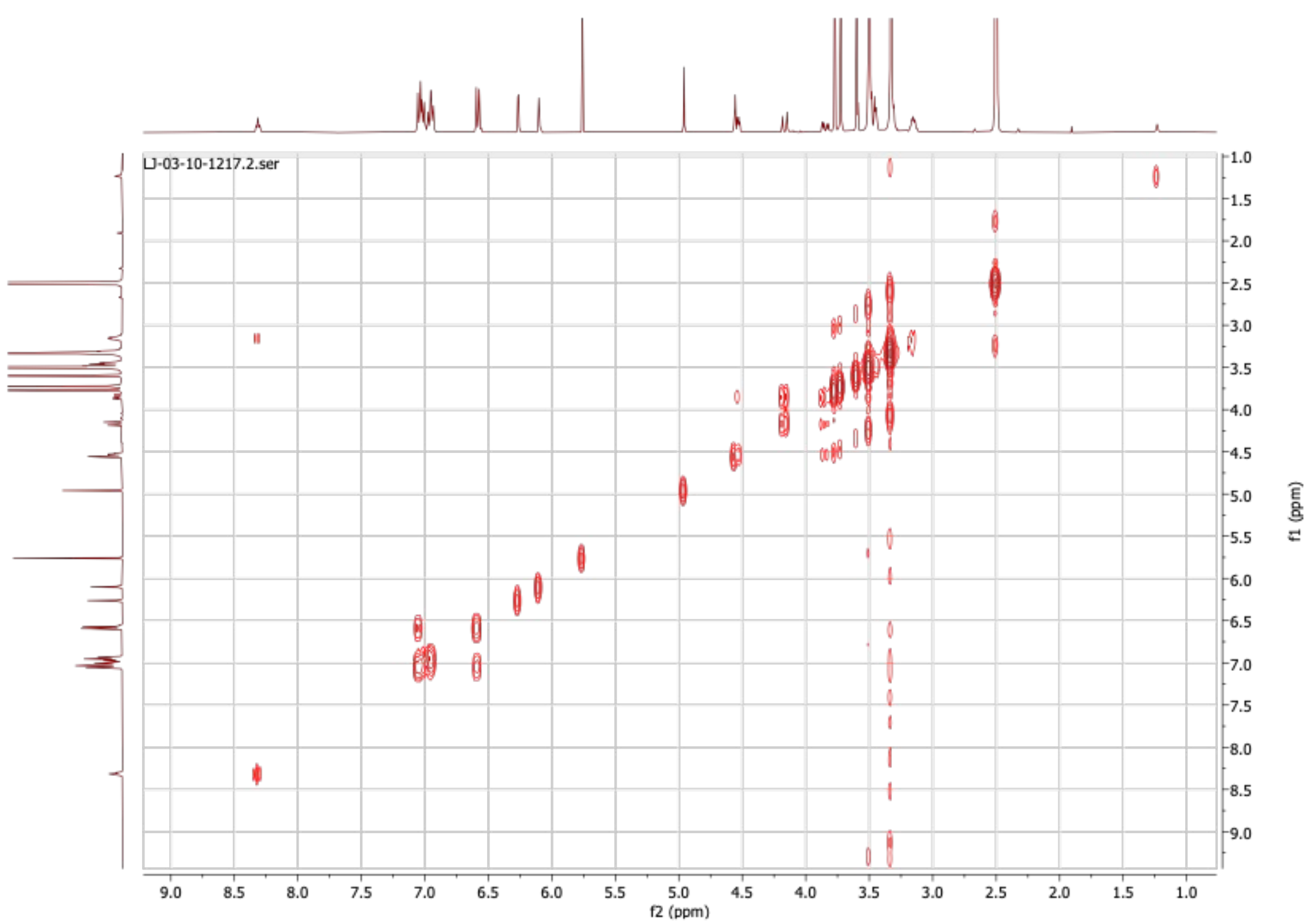

^{1}H NMR and ^{1}H-^{1}H COSY of RocA-PAL

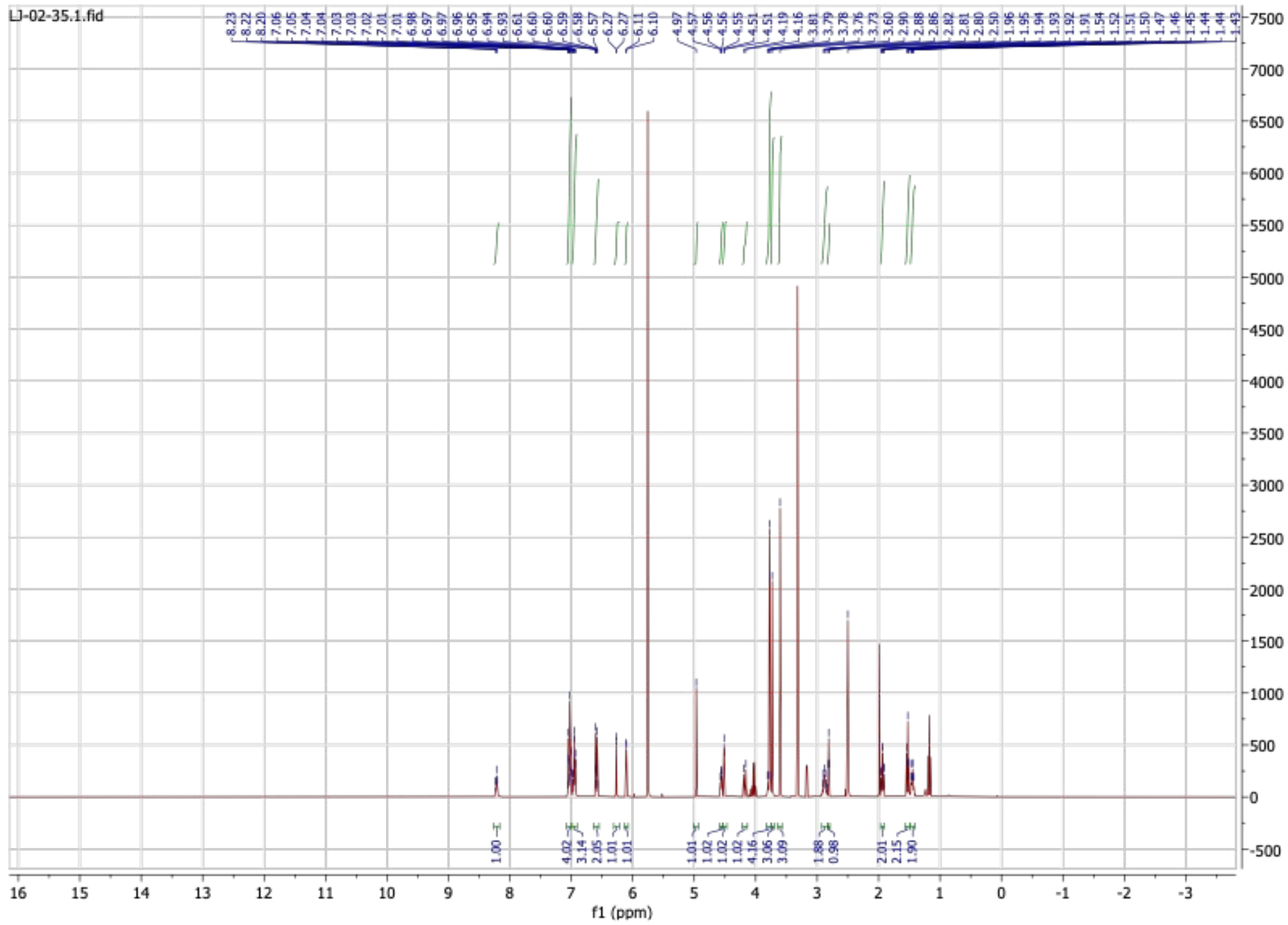

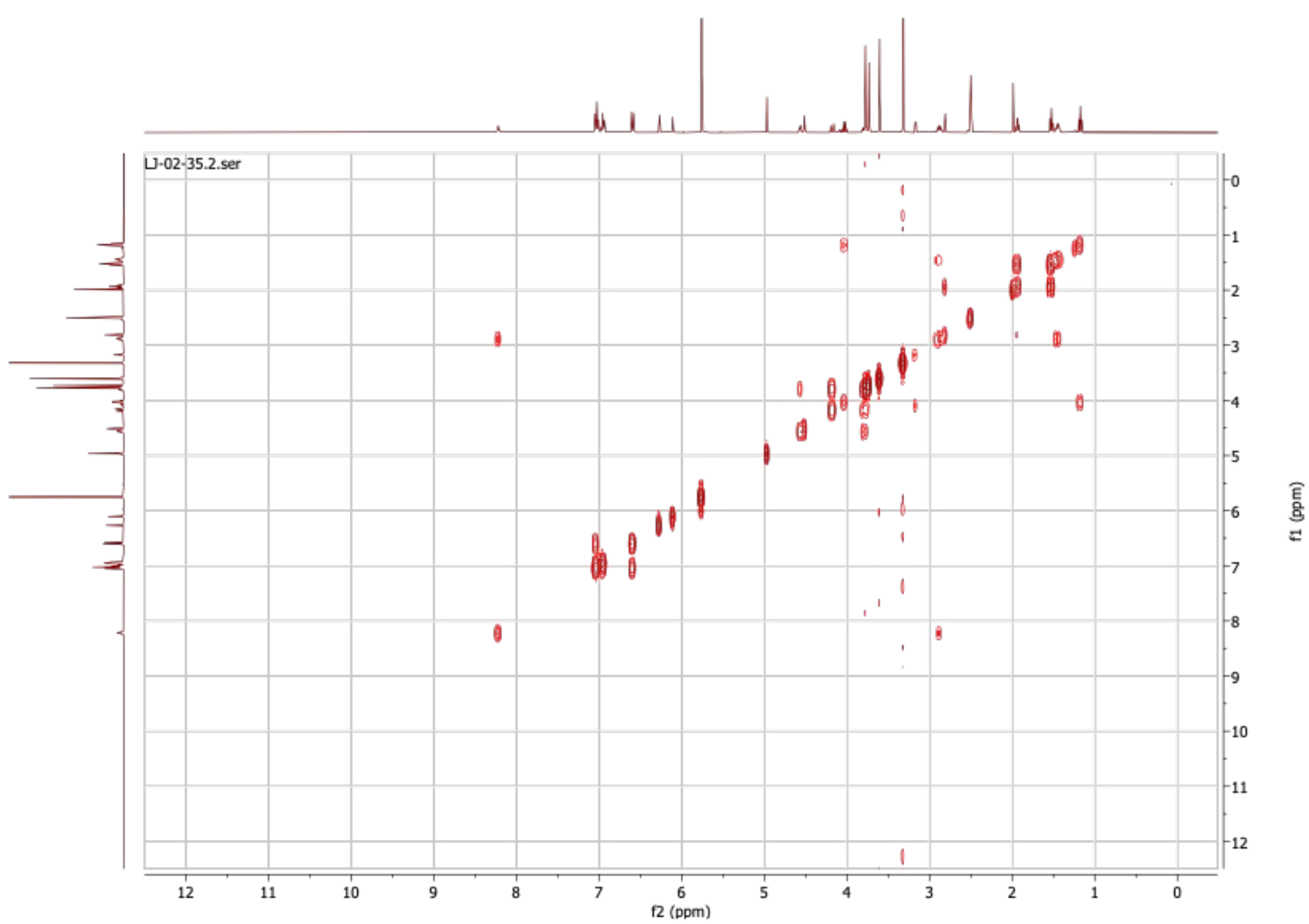

Purity of HemiRoc

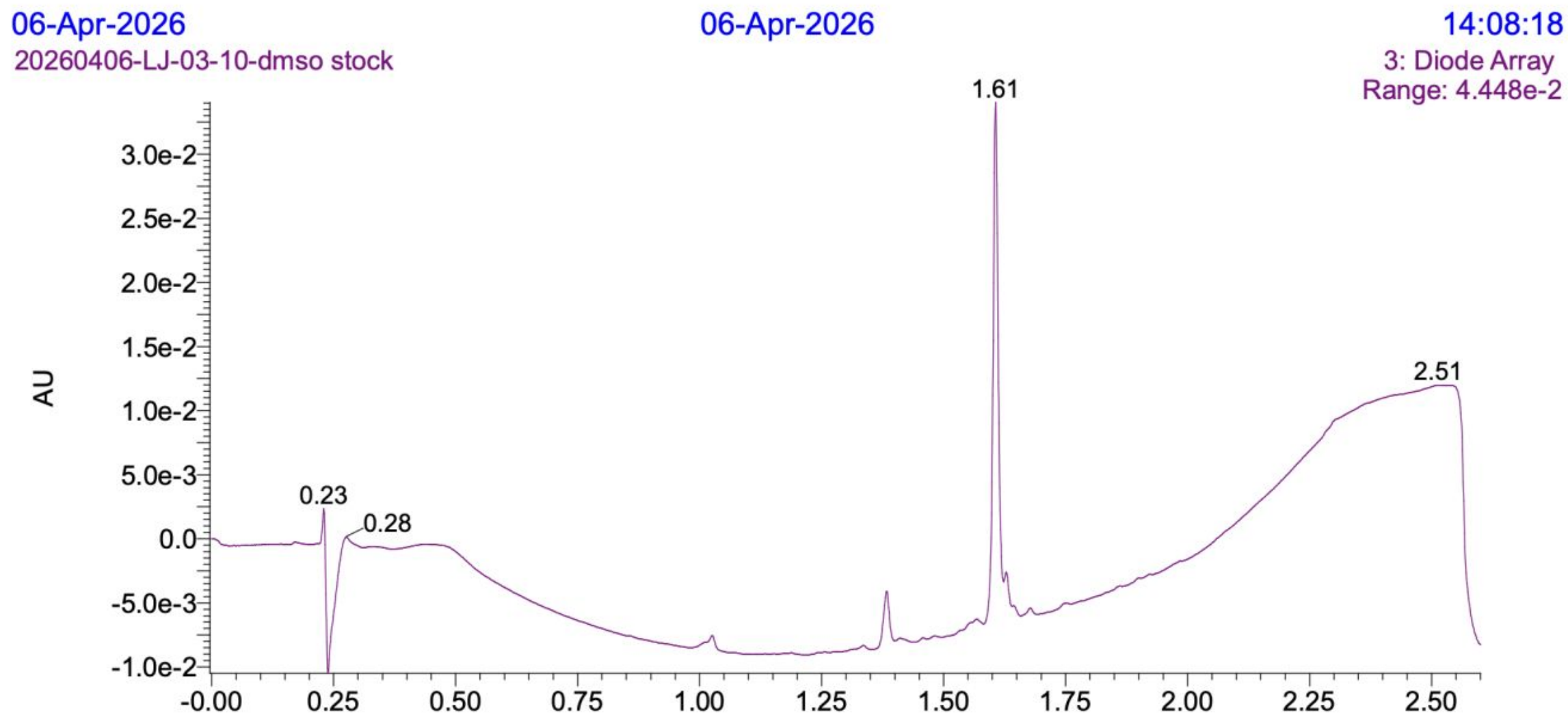

Purity of RocA-PAL

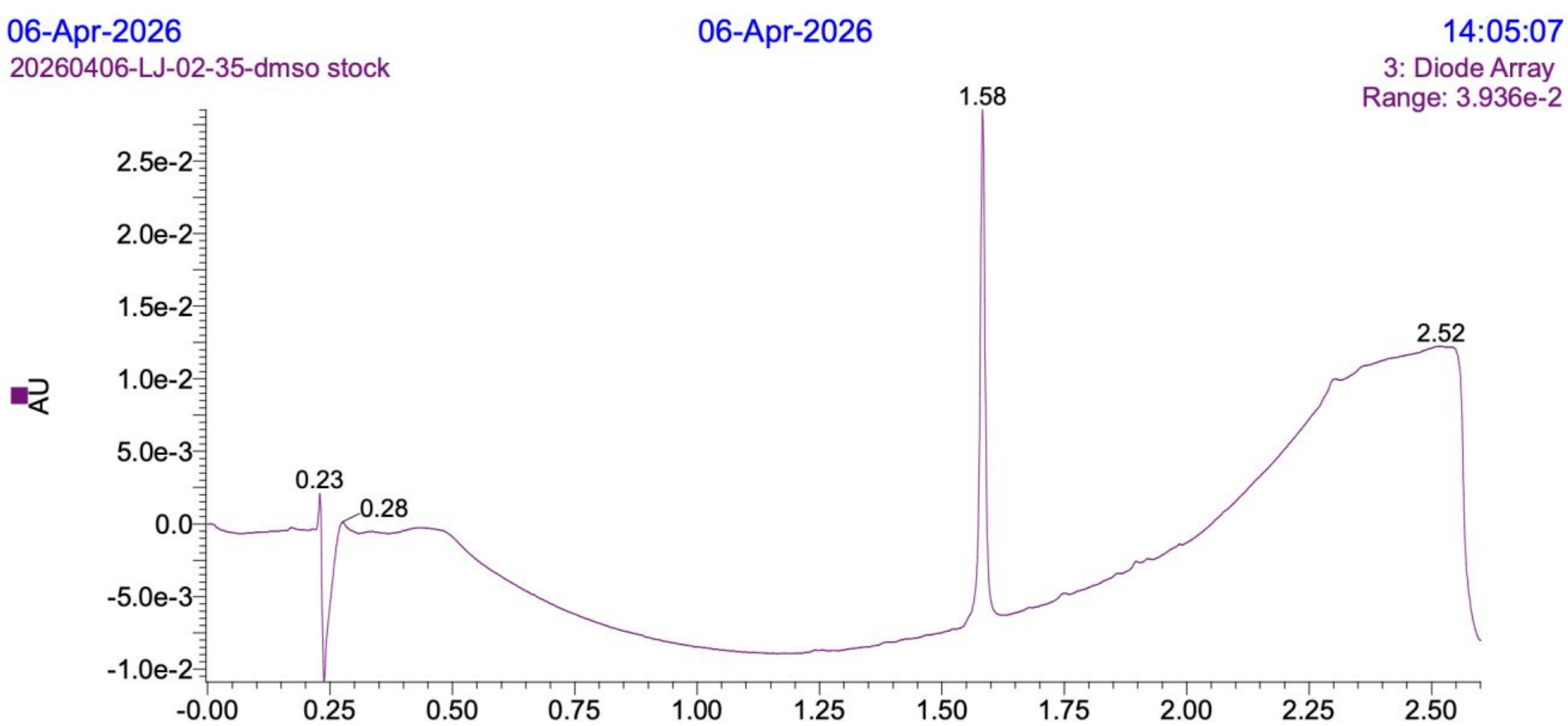

Supplement: Supplementary file 1 [file oc6c00662_si_002.pdf]
